# HDAC1,2 inhibition impairs EZH2- and BBAP- mediated DNA repair to overcome chemoresistance in EZH2 gain-of-function mutant diffuse large B-cell lymphoma

**DOI:** 10.18632/oncotarget.3120

**Published:** 2014-12-31

**Authors:** Danielle P. Johnson, Gabriella S. Spitz, Shweta Tharkar, Steven N. Quayle, Jeffrey R. Shearstone, Simon Jones, Maria E. McDowell, Hannah Wellman, Jessica K. Tyler, Bradley R. Cairns, Mahesh B. Chandrasekharan, Srividya Bhaskara

**Affiliations:** ^1^ Department of Radiation Oncology, Huntsman Cancer Institute, University of Utah School of Medicine, Salt Lake City, UT; ^2^ Department of Oncological Sciences, Huntsman Cancer Institute, University of Utah School of Medicine, Salt Lake City, UT; ^3^ Acetylon Pharmaceuticals, Inc., Boston, MA; ^4^ Department of Biochemistry and Molecular Biology, The University of Texas MD Anderson Cancer Center, Houston, TX

**Keywords:** epigenetics, DNA repair, HDAC1,2, chromatin, EZH2

## Abstract

Gain-of-function mutations in the catalytic site of EZH2 (Enhancer of Zeste Homologue 2), is observed in about 22% of diffuse large B-cell lymphoma (DLBCL) cases. Here we show that selective inhibition of histone deacetylase 1,2 (HDAC1,2) activity using a small molecule inhibitor causes cytotoxic or cytostatic effects in EZH2 gain-of-function mutant (EZH2^GOF^) DLBCL cells. Our results show that blocking the activity of HDAC1,2 increases global H3K27ac without causing a concomitant global decrease in H3K27me3 levels. Our data shows that inhibition of HDAC1,2 is sufficient to decrease H3K27me3 present at DSBs, decrease DSB repair and activate the DNA damage response in these cells. In addition to increased H3K27me3, we found that the EZH2^GOF^ DLBCL cells overexpress another chemotherapy resistance factor − B-lymphoma and BAL-associated protein (BBAP). BBAP monoubiquitinates histone H4K91, a residue that is also subjected to acetylation. Our results show that selective inhibition of HDAC1,2 increases H4K91ac, decreases BBAP-mediated H4K91 monoubiquitination, impairs BBAP-dependent DSB repair and sensitizes the refractory EZH2^GOF^ DLBCL cells to treatment with doxorubicin, a chemotherapy agent. Hence, selective HDAC1,2 inhibition provides a novel DNA repair mechanism-based therapeutic approach as it can overcome both EZH2- and BBAP-mediated DSB repair in the EZH2^GOF^ DLBCL cells.

## INTRODUCTION

Diffuse large B-cell lymphoma (DLBCL), a type of non-Hodgkin's lymphoma, is the most common lymphoid malignancy, accounting for approximately 30% of all adult lymphomas [1]. An optimal treatment for this lymphoma is unavailable at present [2]. Additionally, these lymphoma cells acquire chemoresistance and consequently have a high relapse rate, making it a hard-to-treat disease [1]. Translocations involving *c-Myc*, *BCL2* and *BCL6* are frequently detected in DLBCL patients [3, 4]. Apart from these genetic alterations, recurrent somatic mutations in EZH2 (the H3K27 methyltransferase) have also been identified in DLBCL [5-7]. These mutations occur in tyrosine 641 (Y641) residue within the catalytic SET domain of EZH2, and are found in two types of lymphomas: 21.7% of germinal center-type diffuse large B-cell lymphoma (GC-DLBCL) and 7.2% of follicular lymphoma (FL) [6]. Mutations in EZH2 Y641 are gain-of-function mutations that result in a hyperactive EZH2 catalyzing aberrantly high levels of H3K27 trimethylation (H3K27me3) [5]. H3K27me3, a transcriptional repression mark, is proposed to stably repress tumor suppressor *BLIMP1* expression in GC-DLBCL to contribute to lymphomagenesis [5]. GSK126, a potent and selective inhibitor of EZH2 activity, decreases H3K27me3 to promote cell death in DLBCL cells, especially in the chemoresistant or refractory EZH2 gain-of-function mutant DLBCL cells [8]. A recent study showed a correlation between increased H3K27me3 and chemoresistance in cancer [9]. Therefore, decreasing H3K27me3 in the refractory EZH2 gain-of-function mutant (henceforth referred to as EZH2^GOF^) DLBCL cells with a small molecule inhibitor of EZH2 activity is one strategy to overcome the H3K27me3-mediated resistance to chemotherapy.

Histone deacetylase inhibitors (HDAC inhibitors/HDIs) are potent anticancer drugs [10]. Several broad-spectrum HDIs are in various stages of clinical trials for both solid tumors and hematopoietic malignancies. Two of these compounds (Vorinostat and Romidepsin) have gained FDA approval for use in refractory cutaneous T-cell lymphoma and belinostat was recently approved for use in peripheral T-cell lymphoma. However, a FDA-approved HDI for the treatment of B-cell lymphoma is not yet available [11, 12]. HDAC1 and HDAC2 (henceforth referred to as HDAC1,2) belong to class Ι HDAC family [13] and interact with the polycomb repression complex 2 (PRC2) that contains EZH2 as the catalytic subunit. HDAC inhibition was previously shown to relieve transcriptional repression mediated by PRC2 [14]. We therefore asked whether the compromised viability of the EZH2^GOF^ DLBCL cells achieved by an EZH2 inhibitor can also be obtained using an HDAC1,2-selective inhibitor.

In this study, we investigated the efficacy and the mechanism of action of a HDAC1,2-selective inhibitor (ACY-957) in EZH2^GOF^ DLBCL cells. Using this HDAC1,2-selective inhibitor, we show that loss of HDAC1,2 activity increases global H3K27ac and impairs proliferation of the EZH2^GOF^ DLBCL cells within a short three day treatment. Our studies show that HDAC1,2 activity are critical for the enrichment of H3K27me3 at double-strand break (DSB) sites during DNA repair and loss of HDAC1,2 activity impairs efficient DSB repair in these refractory DLBCL cells. Hence, our findings show how HDAC1,2 inhibition can overcome the high level of repair activity mediated by the aberrantly increased H3K27me3 as a result of a hyperactive EZH2 in the chemoresistant EZH2^GOF^ DLBCL cells. In addition to their role at the DNA break sites, HDAC1,2 inhibition increases H3K27ac globally and at the promoters of DNA damage response genes, suggesting a role for HDAC1,2 in maintaining the H3K27ac-H3K27me3 balance within the cell. We also report that the EZH2^GOF^ DLBCL cells overexpress BBAP, (B-lymphoma and BAL-associated protein), an E3 ligase involved in monoubiquitination of histone H4K91 [15], a factor that was shown to be associated with chemoresistance previously [16-18]. Our findings show that H4K91ac is a novel target of HDAC1,2. We report that HDAC1,2 inhibition decreases H4K91 ubiquitination during DNA repair in response to doxorubicin (a chemotherapy agent), overcomes the BBAP-mediated DNA repair and sensitizes the otherwise chemoresistant or refractory EZH2^GOF^ DLBCL cells to doxorubicin (a chemotherapy agent). Therefore, our studies show that HDAC1,2 activity regulate H4K91 ubiquitination and H3K27me3 during DNA repair in the EZH2^GOF^ DLBCL cells. In summary, our studies show that a single selective inhibitor of HDAC1,2 can overcome the DNA repair and chemoresistance mediated by two chromatin-modifying enzymes − EZH2 (a histone methyltransferase) and BBAP (a histone E3 ubiquitin ligase).

## RESULTS

### H3K27me3 is increased in the EZH2^GOF^ DLBCL cells compared to other cancer cells

**Figure 1:**
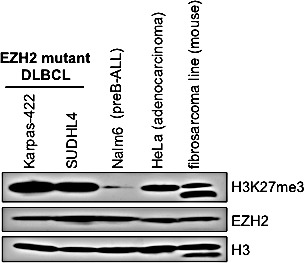
Levels of histone H3K27me3 in EZH2 DLBCL cells when compared to other cancer cell lines Western blot analysis of whole cell lysates prepared from Karpas-422, SUDHL4, NALM6, HeLa and mouse fibrosarcoma cells was performed with anti-H3K27me3 and anti-EZH2 antibodies. Histone H3 served as a loading control.

The Karpas-422 line was established from the pleural effusion of a patient with chemotherapy-resistant non-Hodgkin's lymphoma (NHL) [19] and the SUDHL4 line was derived from the peritoneal effusion of a 38-year male NHL patient [20]. Karpas-422 and SUDHL4 lines express mutant EZH2 with an amino acid substitution in the catalytic SET domain: the Karpas-422 line contains the Y641N mutation and the SUDHL4 line expresses the Y641S mutant [21]. These changes in EZH2 were previously shown to be gain-of-function mutations resulting in a hyperactive enzyme [7]. We examined whether EZH2 catalyzed H3K27 trimethylation (H3K27me3) is augmented in Karpas-422 and SUDHL4 lines compared to other cancer cell lines. Extracts were prepared for Western analysis from Karpas-422, SUDHL4, NALM6 (a pre B-acute lymphoblastic leukemia line) [22], HeLa (a cervical adenocarcinoma line) and Rosa26 (a mouse fibrosarcoma line). Protein levels of EZH2 in Karpas-422 and SUDHL4 lines were similar to that present in other cancer cell lines (Figure 1). However, increased H3K27me3 levels were observed in the Karpas-422 and SUDHL4 lines compared to NALM6, HeLa and mouse fibrosarcoma lines (Figure 1). Therefore, the Y641N or Y641S mutations in the EZH2 SET domain do not affect EZH2 protein levels, but result in a hyperactive enzyme causing aberrantly increased H3K27me3 in the EZH2^GOF^ DLBCL cells compared to other cancer cell lines.

### ACY-957 is a novel selective small molecule inhibitor of HDAC1 and HDAC2 enzymatic activity

**Figure 2:**
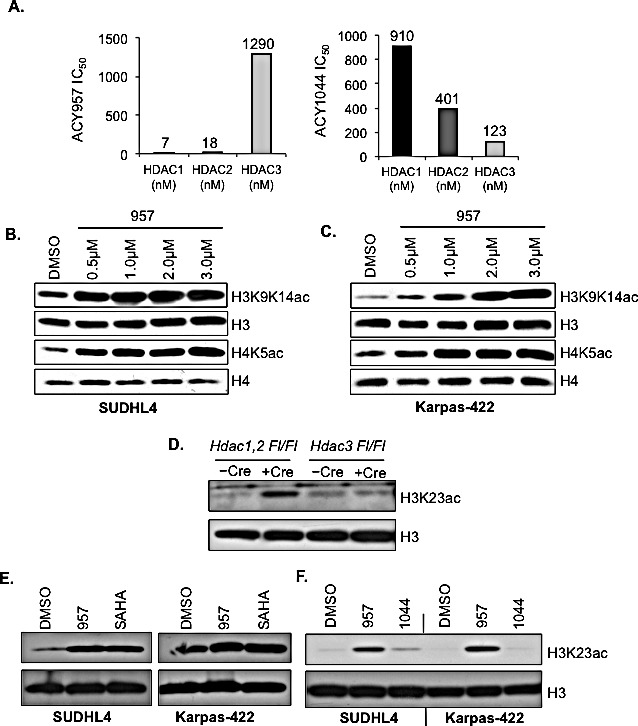
Selective HDAC1,2 inhibition increases H3K27ac in EZH2^GOF^ DLBCL cells A: In vitro enzyme assays using recombinant HDAC proteins to determine the specificity of ACY-957 and ACY-1044 towards HDAC1, 2 and 3. Numbers in the graphs represent IC50 values obtained for an inhibitor-enzyme combination in the in vitro HDAC assays at a 95% confidence level. Compounds were diluted in DMSO to 50 fold the final concentration and a ten point three fold dilution series was made. The compounds were diluted in assay buffer (50 mM HEPES, pH 7.4, 100 mM KCl, 0.001% Tween-20, 0.05% BSA, 20 μM tris(2-carboxyethyl)phosphine) to 6 fold their final concentration. The HDAC enzymes (purchased from BPS Biosciences) were diluted to 1.5 fold their final concentration in assay buffer. The tripeptide substrate (synthesized in house) and trypsin at 0.05 μM final concentration were diluted in assay buffer at 6 fold their final concentration. Five μM compounds and 20 μM of enzyme were added to wells of a black, opaque 384 well plate in duplicate. Enzyme and compound were incubated together at room temperature for 10 minutes. Five l of substrate was added to each well, the plate was shaken for 60 seconds and placed into a Victor 2 microtiter plate reader. The development of fluorescence was monitored for 60 min and the linear rate of the reaction was calculated. The IC50 was determined using Graph Pad Prism by a four parameter curve fit. SUDHL4 (B) and Karpas-422 (C) cells were treated with increasing amounts of ACY-957 and whole cell lysates were prepared following a 24h treatment. Western blot analysis of H3K9K14ac and H4K5ac was done. Histone H3 and H4 were used as controls. D: Western blot analysis of whole cell lysate prepared from *HDAC1^Fl/Fl^, HDAC2^Fl/Fl^* or *HDAC3^Fl/Fl^* fibrosarcoma cells following Ad-Cre infection. Lysates were prepared 72h post Ad-Cre infection. E: SUDHL4 and Karpas-422 cells were treated with DMSO, 2μM ACY-957 or 2μM SAHA for 24h and western blot analysis of whole cell lysate with anti-H3K23ac was done. Histone H3 served as the loading control. F: SUDHL4 and Karpas-422 cells were treated with either DMSO, 2μM ACY-957 or 2μM ACY-1044 and western analysis with anti-H3K23ac was performed.

H3K27me3 is linked to transcriptional repression and it is enriched at the transcription start sites of inactive genes [23]. H3K27me3 has been implicated to contribute to the proliferation and chemoresistance in a subset of DLBCL cells [24]. Inhibition of EZH2 activity in the GC-derived DLBCL cells using a small molecule inhibitor was previously shown to reduce global H3K27me3 levels, induce the expression of BLIMP1 (a tumor suppressor) and impair the *in vitro* growth of lymphoma cells [5]. Therefore, inhibiting the constitutively active enzymatic function of EZH2 in order to reduce the aberrant H3K27 hypertrimethylation is one strategy to overcome lymphomagenesis in DLBCL cells. In addition to methylation, the H3K27 residue also undergoes acetylation, which is dynamically regulated by the action of histone acetyltransferases (HATs) and deacetylases (HDACs). HDAC1,2 interact with the EZH2-containing PRC2 complex [14] and may function to remove the acetyl group from the lysine residue in order for EZH2 catalyzed methylation to occur. Therefore, inhibition of HDAC1,2 activity alone or in combination with inhibition of EZH2 activity could be an alternative strategy to overcome lymphomagenesis in DLBCL cells by blocking the gain-of-function mutant EZH2 mediated H3K27 hypertrimethylation via increased histone acetylation. To test this possibility, we chose to inhibit HDAC1,2 activity in DLBCL cells using a novel small molecule, ACY-957. Among class I HDACs, HDAC1,2 share a 50% sequence homology with HDAC3 [25]. Therefore, we first determined the selectivity of ACY-957 towards HDAC1 and HDAC2 in *in vitro* enzyme assays using recombinant enzymes. We also included ACY-1044, a selective small molecule inhibitor of HDAC3, as a control in our characterization studies. The biochemical IC_50_ values obtained using *in vitro* HDAC assays showed that a 7.4-fold and 3.3-fold higher concentration of ACY-1044 (the HDAC3-selective inhibitor) was required to inhibit HDAC1 and HDAC2, respectively, when compared to HDAC3 (Figure 2A). In contrast, ACY-957 was 185-fold selective for HDAC1 and 72-fold more selective for HDAC2 when compared to HDAC3 (Figure 2A).

Next, we tested the ability of these small molecules to inhibit HDAC1,2 or HDAC3 activities *in vivo* in EZH2^GOF^ DLBCL cell lines. We treated Karpas-422 and SUDHL4 cells with increasing amounts of ACY-957 for 24h and examined changes in histone acetylation marks; specifically, acetylation at histone H3 K9 and K14 residues (H3K9,K14ac) and at histone H4 K5 residue (H4K5ac), which are increased in *Hdac1,2^−/−^* cells [26-28]. Western analysis showed a concentration-dependent increase in the levels of H3K9,K14ac and H4K5ac in ACY-957 treated Karpas-422 and SUDHL4 cells compared to the DMSO (vehicle) treated control cells (Figures 2B and 2C). H3K9,K14ac and H4K5ac levels are also increased in *Hdac3^−/−^* cells [29-32] and treatment of Karpas-422 and SUDHL4 cells with increasing concentrations of ACY-1044 (the HDAC3 inhibitor) also resulted in elevated H3K9,K14ac and H4K5ac levels compared to the DMSO treated control cells (Supplementary Figure 1). However, increase in H3K9,K14ac and H4K5ac levels appeared to be more robust following inhibition of HDAC1,2 activity using ACY-957 than inhibition of HDAC3 activity using ACY-1044 (Figures 2B, 2C and Supplementary Figure 1). Collectively, these results determined the minimum concentration of ACY-957 and ACY-1044 required to inhibit HDAC1,2 or HDAC3 activities *in viv*o in DLBCL cells.

Next we set out to examine the ability of ACY-957 to inhibit only HDAC1,2 activity in DLBCL cells. We performed a screen in *Hdac1,2* or *Hdac3* knockout cells using Western blotting to identify a histone acetylation mark that is targeted by HDAC1,2 and not HDAC3. Western analysis of histone acetylation marks altered in *Hdac1,2^−/−^* or *Hdac3^−/−^* fibrosarcoma cells revealed that global H3K23ac levels are increased only upon loss of HDAC1,2 and not in cells lacking HDAC3 (Figure 2D). Therefore, this result identified H3K23ac as a substrate that is specifically targeted by HDAC1,2 and not HDAC3. Hence, we used changes in H3K23ac as a readout to test the ability of ACY-957 to specifically inhibit HDAC1,2 activity in DLBCL cells. We treated the refractory DLBCL cells, Karpas-422 and SUDHL4, with 2μM ACY-957, 2μM ACY-1044 (the HDAC3 inhibitor) or 2μM Vorinostat (SAHA, a pan HDAC inhibitor that targets HDAC1,2 and HDAC3). Western analysis showed that ACY-957 treatment of DLBCL cells increased H3K23ac levels (Figure 2E). Moreover, increase in H3K23ac obtained using ACY-957 treatment was similar to that obtained following treatment with SAHA (Figure 2E), suggesting that HDAC1,2 are the primary class I HDACs involved in deacetylating H3K23ac. Importantly, addition of ACY-957, but not ACY-1044, to Karpas-422 or SUDHL4 cells resulted in an increase in H3K23ac levels compared to the DMSO treated control cells (Figure 2F). Taken together, these results demonstrated that ACY-957 specifically inhibits HDAC1,2 activity in DLBCL cells.

### Selective inhibition of HDAC1,2 activity causes cytotoxic or cytostatic effects in EZH2^GOF^ DLBCL cells

Having established that ACY-957 is a selective inhibitor of HDAC1,2, we next asked whether HDAC1,2 activity are required for the proliferation and/or survival of the EZH2^GOF^ DLBCL cells. Therefore, we treated Karpas-422 and SUDHL4 cells with ACY-957 for 24h, 48h and 72h, and performed cell cycle analysis to measure the extent of cell death (cytotoxic) and/or cell cycle arrest (cytostatic) triggered as a result of inhibiting HDAC1,2 activity in these cells. Since these DLBCL cells express a hyperactive form of EZH2, pharmacological inhibition of EZH2 activity is considered a viable therapeutic strategy. Indeed, several small molecule inhibitors of EZH2 activity with varying modes-of-action are available: GSK126 is a S-adenosyl-methionine-competitive inhibitor of EZH2 methyltransferase activity, and DZNep is a S-adenosylhomocysteine hydrolase inhibitor that disrupts the components of the PRC2 complex causing reduced chromatin-associated EZH2 levels [33]. Therefore, we also treated Karpas-422 and SUDHL4 cells with an EZH2 inhibitor (DZNep or GSK126), either alone or in combination with ACY-957, for the indicated time prior to cell cycle analysis.

**Figure 3:**
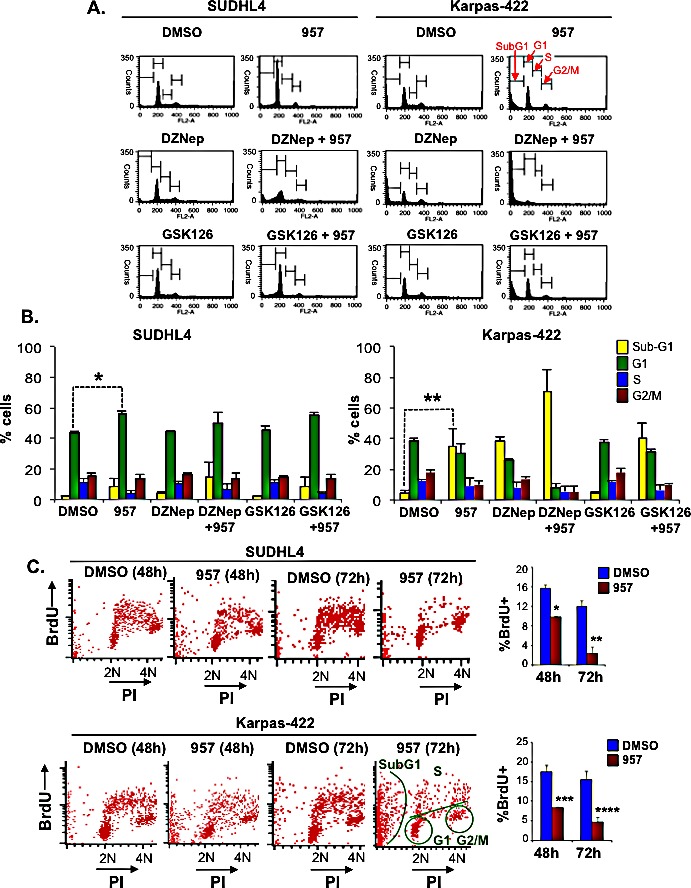
HDAC1,2 inhibition causes apoptosis in Karpas-422 cells and a G1 arrest in SUDHL4 cells A: SUDHL4 and Karpas-422 cells were treated with DMSO, 2μM ACY-957, 0.5μM DZNEP, 0.5μM GSK126 or a combination of drugs for 72h prior to cell cycle analysis by propidium-iodide staining. Representative plots from one experiment out of three independent experiments is shown in the figure. B: Quantitation of the cell cycle data in (A), compiled from three independent analyses. The graphs plot the average percentage of cells in each phase of the cell cycle. Error bars indicate to the standard error calculated from three independent experiments. *p=0.005; **p=0.0176. C: FACS analysis following BrdU-PI staining of Karpas-422 and SUDHL4 cells was performed following a 48h or 72h treatment with ACY-957. Representative plots from two independent experiments are shown in the figure. Quantitation of S-phase cells at 48h and 72h post ACY-957 treatment is also shown in the figure. *p= 0.01, **p=0.03, ***p= 0.03 and ****p= 0.02.

Compared to treatment with ACY-957 for 24h or 48h, treatment of Karpas-422 cells with ACY-957 for 72h resulted in a larger increase in the number of sub-G1/dead cells, and treatment of SUDHL4 cells with ACY-957 for 72h resulted in a greater number of cells arrested in G1 phase and those present in the sub-G1 population (Figures 3A, 3B). Treatment with DZNep alone triggered a modest increase in dead/sub-G1 SUDHL4 cells, but resulted in a larger increase in the number of dead Karpas-422 cells similar to that obtained with ACY-957 treatment (Figure 3A, 3B). Combined addition of both DZNep and ACY-957 caused an enhanced cytotoxic effect with a significant increase in the number of sub-G1 or dead Karpas-422 cells, which is greater than that obtained when these cells were treated with either DZNep alone or ACY-957 alone (Figure 3B). Combined addition of DZNep and ACY-957 to SUDHL4 cells also elicited a similar enhanced cytotoxic effect, albeit a more modest increase in the number dead or sub-G1 cells that is slightly larger than that obtained with ACY-957 or DZNep alone was observed (Figure 3B). Addition of GSK126 alone did not result in any defect in the cell cycle progression and/or survival of Karpas-422 and SUDHL4 cells even after an incubation period of 72h (Figure 3). This result agrees well with the reported finding that GSK126 decreases the viability of Karpas-422 cells only after treatment for 7 days [8]. Since, we see a significant cell death with ACY-957 by 72h, we could not test the synergistic effect of ACY-957 and GSK-126 following a 7-day treatment. Treatment of Karpas-422 and SUDHL4 cells for 24h with the HDAC1,2 selective inhibitor (ACY-957) alone or the EZH2 inhibitor alone (DZNep or GSK126) or their combined addition (DZNep+ACY-957 or GSK126+ACY-957) did not affect their progression through the cell cycle, as the profiles for the inhibitor-treated cells looked similar to that obtained for the DMSO-treated control cells (Supplementary Figure 2). Following 48h incubation, ACY-957 treatment resulted in the accumulation of SUDHL4 cells in G1 phase (G1 arrest) (Supplementary Figure 2A) and a modest accumulation of sub-G1 population (indicative of dead cells) was observed in ACY-957-treated Karpas-422 cells when compared to the control DMSO-treated cells (Supplementary Figure 2B). Inhibition of EZH2 activity using GSK126 alone did not cause any change in the progression of Karpas-422 or SUDHL4 cells through the cell cycle (Supplementary Figure 2). These results suggest that selective inhibition of HDAC1,2 activity causes a more rapid adverse effect on the proliferation and survival of the chemoresistant DLBCL cells than the selective inhibition of EZH2 activity. Treatment with the EZH2 inhibitor DZNep for 48h caused a modest increase in the number of dead Karpas-422 cells similar to that observed with the ACY-957 treatment (Supplementary Figure 2B). However, DZNep when combined with ACY-957 resulted in a significant/substantial increase in the number of sub-G1 or dead Karpas-422 cells (Supplementary Figure 2). No such synergistic effect was observed when ACY-957 was combined with GSK126. These findings together suggest that inhibition of HDAC1,2 activity along with the dissociation of the PRC2 complex using DZNep, but not inhibition of EZH2 activity using GSK126, causes a synergistic cytotoxic effect on Karpas-422 cells. FACS analysis following bromodeoxyuridine (BrdU)-propidium iodide labeling and staining was performed at 48h and 72h following treatment of cells with ACY-957. A decrease in S-phase population accompanied by either increased cell death or a G1 arrest was observed in Karpas-422 and SUDHL4 cells, respectively (Figure 3C). Collectively, results from our analysis of the chemoresistant DLBCL cells showed that HDAC1,2 activity and an intact PRC2 complex are required for progression through the cell cycle and/or survival.

### H3K27ac is robustly increased in the EZH2^GOF^ DLBCL cells upon selective inhibition of HDAC1,2 activity

**Figure 4:**
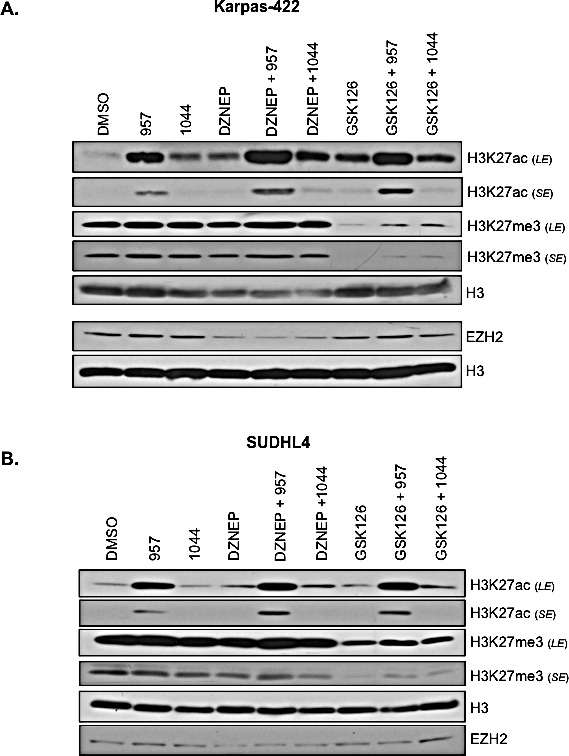
Selective HDAC1,2 inhibition increases global chromatin-associated H3K27ac without altering H3K27me3 in EZH2 DLBCL cells Karpas-422 (A) and SUDHL4 (B) were treated with DMSO, 2μM ACY-957, 2μM ACY-1044, 0.5μM DZNEP, 0.5μM GSK126 or combinations of these drugs for 48h prior to chromatin extraction. Western blot analysis was done with anti-H3K27ac, H3K27me3 or EZH2 and histone H3 served as a loading control. *LE* and *SE* indicate long and short exposures, respectively.

The histone H3K27 residue is subjected to reversible acetylation and methylation. The aberrantly increased H3K27me3 resulting from a hyperactive EZH2 in DLBCL cells is proposed to contribute to both lymphomagenesis and chemoresistance. Hence, we next asked whether the cytotoxic or cytostatic effects of ACY-957 or DZNep on the chemoresistant DLBCL cells correlate with any change in the dynamics of acetylation and methylation at the H3K27 residue on chromatin. Therefore, we treated the refractory DLBCL cells, Karpas-422 and SUDHL4, with DMSO (vehicle), the HDAC1,2 selective inhibitor alone (ACY-957), the EZH2 inhibitor alone (DZNep) or a combination of both these inhibitors (ACY-957+DZNep). We also treated cells with the second EZH2 inhibitor, GSK126 alone, or combined with ACY-957 (ACY-957+GSK126). As an additional control, cells were treated with ACY-1044 (the HDAC3 selective inhibitor) alone or combined with one of the two EZH2 inhibitors (ACY-1044+DZNep or ACY-1044+GSK126). Cells were treated for 48h prior to preparing chromatin extracts for Western analysis using antibodies recognizing H3K27ac or H3K27me3. As shown in Figure 4, selective inhibition of HDAC1,2 activity alone using ACY-957 or when combined with EZH2 inhibitors led to a robust increase in global H3K27ac levels in both Karpas-422 and SUDHL4 cells. Surprisingly, global H3K27me3 levels were not reduced and remained unchanged in ACY-957 treated cells compared to the DMSO treated control cells (Figure 4). While treatment with GSK126 led to a decrease in global H3K27me3 levels as expected, a greater reduction in H3K27me3 levels due to combined action of GSK126 and ACY-957 was not observed (Figure 4). In fact, a slight increase in H3K27me3 levels was observed when cells were treated with both GSK126 and ACY-957 (Figure 4). These results suggest that addition of acetyl groups onto unmodified H3K27 residues occurs on chromatin without requiring the removal of existing H3K27me3. GSK126 treatment caused a significant reduction in global H3K27me3 (Figure 4), but it did not trigger death in the chemoresistant DLBCL by 72h, except in the case of combination with ACY-957 in Karpas-422 cells (Figure 3). In contrast, inhibition of HDAC1,2 activity using ACY-957 and/or reducing chromatin-bound EZH2 using DZNep compromised the viability of the chemoresistant DLBCL without reducing global H3K27me3 levels (Figures 3, 4).

Treatment of SUDHL4 or Karpas-422 cells with a low 0.5μM DZNep decreases global EZH2 protein levels, but it does not decrease global H3K27me3 levels (Figure 4). We have found that only a high 10μM dose of DZNep decreases both global EZH2 and H3K27me3 levels in Karpas-422 cells (data not shown). Our observation that a low dose of DZNep reduces EZH2 protein level without decreasing global H3K27me3 levels is consistent with the findings from several independent studies. Low dose DZNep treatment did not affect bulk H3K27me3 levels in multiple myeloma and lung cancer cell lines [34, 35]. Additionally, 0.2-1μM DZNep treatment of K562 erythroid cells for 72h reduced cell viability, but global level of H3K27me3 remained unaffected even though Western blotting showed reduced EZH2 levels [36]. However, in this study, it was shown that DZNep treatment decreases H3K27me3 at select loci, such as, *SLC4A1* and *EPB42*, which are targets of the ETO2 co-repressor. Based on all these studies, we can surmise that the bulk H3K27me3 levels appears to be relatively stable, and not subjected to dynamic regulation. However, at select loci, active H3K27me3 catalyzed by EZH2, either because of demethylation or independent of demethylation, might be required to regulate nuclear processes, such as, gene transcription. It is conceivable that these loci are therefore in particular sensitive to low DZNep treatment, which is sufficient to promote the degradation of EZH2-containing PRC2 complex. Absence of EZH2 thus leads to the loss of active H3K27me3 at these loci, which could include genes controlling cell viability. Overall, our results together suggest that the viability and/or cell cycle progression of the refractory DLBCL cells is dependent on HDAC1,2 activity and an intact EZH2 containing PRC2 complex but not on global H3K27me3. This is consistent with elegant previous studies in which DZNep caused depletion of PRC2 complex proteins in AML cells [37].

### Selective inhibition of HDAC1,2 induces the expression of DNA damage response genes in EZH2^GOF^ DLBCL cells

While selective inhibition of HDAC1,2 activity does not reduce global H3K27me3 in the EZH2^GOF^ cells (Figure 4), it is conceivable that the cell cycle arrest and/or death triggered in these cells by HDAC1,2 inhibition might be due to increased H3K27ac with a concomitant decrease in H3K27me3 locally at select target loci, such as, genes involved in cell cycle regulation, DNA repair or DNA damage signaling and apoptosis, which could also be the targets of EZH2. To test this possibility, we examined changes in gene expression in Karpas-422 cells following ACY-957 treatment. Three independent DMSO or ACY-957 treatments of Karpas-422 cells for 24h was performed for total RNA isolation, which was then subjected to RNA-seq involving next-generation sequencing and bioinformatics analysis. Our RNA-seq analysis showed that expression of 492 genes was up regulated following inhibition of HDAC1,2 activity in Karpas-422 cells compared to the DMSO control (Supplementary Figure 3A). In order to determine whether genes up regulated following inhibition of HDAC1,2 activity are also targets of EZH2, we compared our gene set with GSK-126 treated Karpas-422 RNA-seq dataset reported by Bradley et al. (2014) [38]. In this published work, target genes of EZH2 were identified by determining changes in the expression following treatment of Karpas-422 cells with GSK-126. This comparison yielded a list of 71 genes that were commonly up regulated in both the datasets (Supplementary Figure 3A). A similar comparison yielded 6 targets that were common in the list of genes down regulated following inhibition of HDAC1,2 activity or upon inhibition of EZH2 (Supplementary Figure 3A). Classification of the common set of 71 genes that were up regulated in ACY-957 treated samples and the known EZH2 target genes based on their cellular functions revealed that they belong to pathways, such as, signaling and are not related to apoptosis, DNA repair, DNA damage response and cell cycle regulation (Supplementary Figure 3B). Inhibition of EZH2 activity is proposed to inhibit tumor growth in non-Hodgkin's lymphoma via inducing the expression of *BLIMP1* (a tumor suppressor) [8]. However, DESeq analysis of our RNA-seq data showed that *BLIMP1* expression was modestly up regulated following selective inhibition of HDAC1,2 (1.7-fold). GSK-126 treatment of Karpas-422 cells led to a 2.4-fold increase in Karpas-422 cells [38]. However, we saw robust cell death with ACY-957 when compared to GSK-126 even when a high concentration of 2μM GSK-126 was used on these cells (data not shown). Hence, up regulation of *BLIMP1* alone is unlikely to be the major cause of cell death in the chemoresistant Karpas-422 cells upon ACY-957 treatment. Taken together, these findings suggest that inhibition of HDAC1,2 activity impinges on the viability and cell cycle progression of refractory DLBCL cells likely by affecting processes other than EZH2 regulated gene expression.

When cells sense endogenous DNA damage, BMF (*BCL2-modifying factor*, a pro-apoptotic gene) promotes apoptosis by binding to the anti-apoptotic BCL2 protein [39]. SAHA, a pan-HDAC inhibitor, was previously shown to induce tumor cell selective expression of the *BMF* gene [40]. We found that BMF expression is increased by 2.5-fold upon selective inhibition of HDAC1,2 activity (Supplementary Figure 3C). Additional search for genes related to the DNA damage response up regulated following inhibition of HDAC1,2 activity revealed a 2-fold increase in the expression of *TP63* (tumor protein 63), a p53 family member (Supplementary Figure 3C). TP63 is required for p53-dependent apoptosis in mouse embryo fibroblasts (MEFs) following endogenous DNA damage [41]. To gain insight into the mechanism of transcriptional activation at the *TP63* and *BMF* genes following inhibition of HDAC1,2 activity, we examined whether changes, if any, occur in the levels of H3K27ac (an activating mark) and H3K27me3 (a repressive mark) at the regulatory promoter regions of these genes using chromatin immunoprecipitation (ChIP) assay. Real-time PCR analysis of ChIP DNA showed an increase in H3K27ac levels at the promoter regions of both *TP63* and *BMF* genes following ACY-957 treatment (Supplementary Figure 3D). However, a concomitant decrease in H3K27me3 was not observed at the *TP63* and *BMF* promoters (Supplementary Figure 3D). To determine whether HDAC1 and HDAC2 play a direct role at these genes and regulate H3K27ac at their promoter regions, we performed ChIP analysis with HDAC1 and HDAC2 antibodies. We found a significant enrichment in HDAC1 and HDAC2 occupancies at the *BMF* and *Tp63* promoters (Supplementary Figure 3E). These results suggest that HDAC1,2 inhibition triggers death and cell cycle defects in the refractory DLBCL cells without requiring a reduction in the repressive H3K27me3 at the target genes. Overall, data from our gene expression analyses allude to a possibility that the death and/or cell cycle arrest in chemoresistant/refractory DLBCL cells triggered by selective inhibition of HDAC1,2 activity is from the activation of DNA damage response potentially resulting from endogenous DNA breaks.

### Selective inhibition of HDAC1,2 activity increases DNA damage and impairs DNA repair in the EZH2^GOF^ DLBCL cells

**Figure 5:**
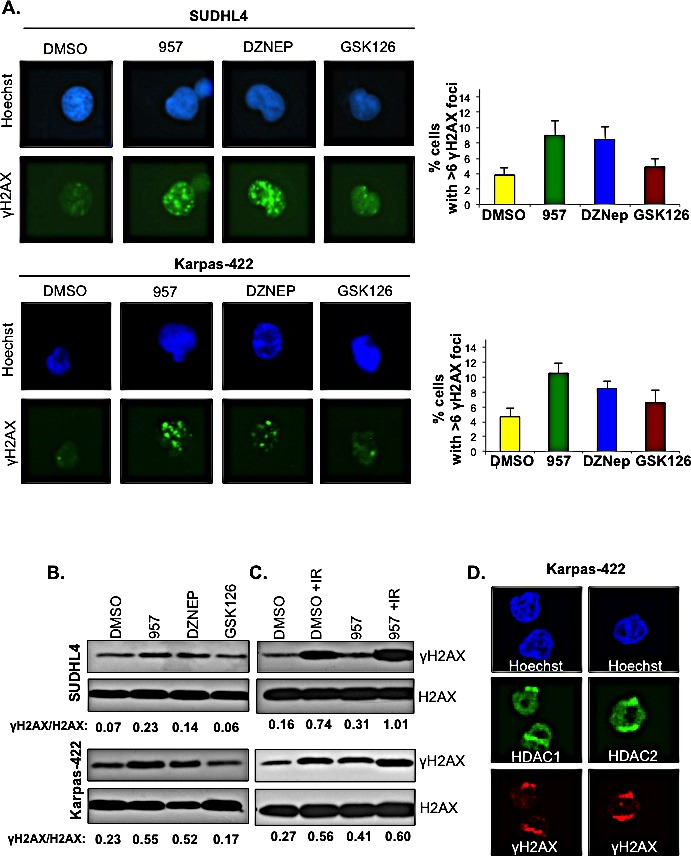
Selective inhibition of HDAC1,2 activates DNA damage response and impairs DSB repair in EZH2 DLBCL cells A: SUDHL4 and Karpas-422 cells were treated with DMSO, 2μM ACY-957, 0.5μM DZNEP or 0.5μM GSK126 for 48h and immunofluorescence staining with γH2AX was performed. The percentage of cells with 6 or greater γH2AX foci were counted in three independent experiments and at least 100 cells were counted in each experiment. The average with standard errors calculated from three independent experiments is shown in the figure. B: SUDHL4 and Karpas-422 cells were treated with DMSO, 2μM ACY-957, 0,5μM DZNEP or 0.5μM GSK126 for 48 h. Histones were purified using trichloroacetic acid extraction protocol (see methods). Western blot analysis was done with anti-γH2AX and histone H2AX served as the loading control. C: SUDHL4 and Karpas-422 cells were treated with DMSO or 2μM ACY-957 for 48h. Following DMSO or ACY-957 treatment, cells were exposed to a 5Gy dose of ionizing radiation and allowed to recover for 30 minutes prior to chromatin extraction. Western blot analysis was done with anti-γH2AX where total histone H2AX served as a loading control. D: Karpas-422 cells were micro-irradiated with laser and allowed to recover for 15 minutes before fixation and immunofluorescence staining with anti-γH2AX, anti-HDAC1 and anti-HDAC2 antibodies was done.

Next, we tested whether selective inhibition of HDAC1,2 activity in EZH2^GOF^ DLBCL cells causes endogenous DNA breaks. Serine 139 of H2AX is phosphorylated (γH2AX) in response to DNA breaks and it accumulates on chromatin over many megabases around a break site to form the nuclear foci [42]. γH2AX foci formation is considered a marker of DNA damage and is used to measure the production of DNA damage and the subsequent repair of the DNA lesion. We measured γH2AX foci formation using immunofluorescence in SUDHL4 and Karpas-422 cells, following a 24h or 48h treatment with DMSO or ACY-957. DZNep and GSK126, the EZH2 inhibitors, were also included in this analysis for comparison. Following a 24h treatment, no significant increase in DNA damage was observed in EZH2^GOF^ DLBCL cells treated with the HDAC1,2 selective inhibitor or the EZH2 inhibitors (data not shown), which agrees well with the lack of any defects in the cell cycle progression observed at this time point using these inhibitors (Supplementary Figure 2). A significant increase in the number of cells with greater than six γH2AX foci was observed upon ACY-957 or DZNep treatment of SUDHL4 and Karpas-422 cells for 48h (Figure 5A). GSK126 treatment caused a slight increase in γH2AX foci at this time point (Figure 5A). We confirmed our results from immunofluorescence by examining changes in γH2AX levels using Western analysis of acid extracted samples prepared following treatment of SUDHL4 and Karpas-422 cells with ACY-957 or either of the EZH2 inhibitors. Similar to that observed using immunofluorescence, ACY-957 treatment led to an increase in γH2AX compared to the control DMSO treatment in both SUDHL4 and Karpas-422 cells (Figure 5B). Between the two EZH2 inhibitors, DZNep treatment caused a greater increase in γH2AX levels compared to GSK126 (Figure 5B), consistent with the observations using immunofluorescence (Figure 5A). These results suggest that both HDAC1,2 and EZH2 activities are required to prevent the accumulation of endogenous DNA damage.

Increase in γH2AX and persistent endogenous DNA damage might occur as a consequence of impaired DNA repair. Therefore, to measure the efficiency of DNA repair, we examined γH2AX levels on chromatin after a recovery period when DNA repair occurs following exposure to an exogenous DNA damaging agent. The EZH2^GOF^ DLBCL cells, SUDHL4 and Karpas-422, were treated with DMSO or ACY-957 for 48h prior to exposure to ionizing radiation (IR). Chromatin extracts were prepared from these cells following a 30 min recovery after exposure to IR and analyzed by Western blotting. γH2AX levels on chromatin in the ACY-957-treated cells post-recovery from IR were more than those present on chromatin in the control DMSO-treated cells post-recovery from IR and in the control cells treated with either DMSO or ACY-957 without any exposure to IR (Figure 5C). These results indicate that HDAC1,2 activity is required for efficient DNA repair and that inhibition results in the persistence of DNA breaks. Collectively, our results suggest that selective inhibition of HDAC1,2 in the EZH2^GOF^ DLBCL impairs DNA repair and activates the DNA damage response.

### HDAC1,2 activity are required for the enrichment of H3K27me3 at the break sites during DNA repair in EZH2^GOF^ DLBCL cells

We next set out to gain insights into mechanism by which selective inhibition of HDAC1,2 activity impairs DNA repair in the chemoresistant EZH2^GOF^ DLBCL cells. As shown in Figure 5, both HDAC1,2 and EZH2 activities are crucial for the repair of DNA breaks. EZH2 and H3K27me3 also localize to the sites of DNA damage and are suggested to play a role in the repair of double strand DNA breaks [43]. HDAC1 and HDAC2 localize to DNA break sites in Karpas-422 cells (Figure 5D), as evidenced in other cell types [44]. Therefore, we asked whether inhibiting HDAC1,2 activity impairs DNA repair in the refractory DLBCL cells by altering the acetylation-methylation dynamic at the H3K27 residue on chromatin. We first examined whether inhibiting HDAC1,2 activity induces any change in global H3K27ac or H3K27me3 on chromatin following DNA damage and during DNA repair. SUDHL4 or Karpas-422 cells with or without exposure to ionizing radiation (IR) were initially treated with DMSO or ACY-957 for 48h. Chromatin extracts were prepared from these cells following a 30 min recovery period (the repair phase) and changes in histone modifications were examined by Western blotting. Global H3K27ac levels on chromatin were increased in ACY-957 treated DLBCL cells compared to the control DMSO treated cells with or without exposure to IR (Supplementary Figure 4A). However, a concomitant decrease in global H3K27me3 levels on chromatin was not observed following the 30 min recovery period from IR in the ACY-957 treated cells compared to the DMSO treatment (Supplementary Figure 4A). These results suggest that HDAC1,2 actively remove H3K27ac on chromatin during DNA repair without affecting global H3K27me3 levels.

**Figure 6:**
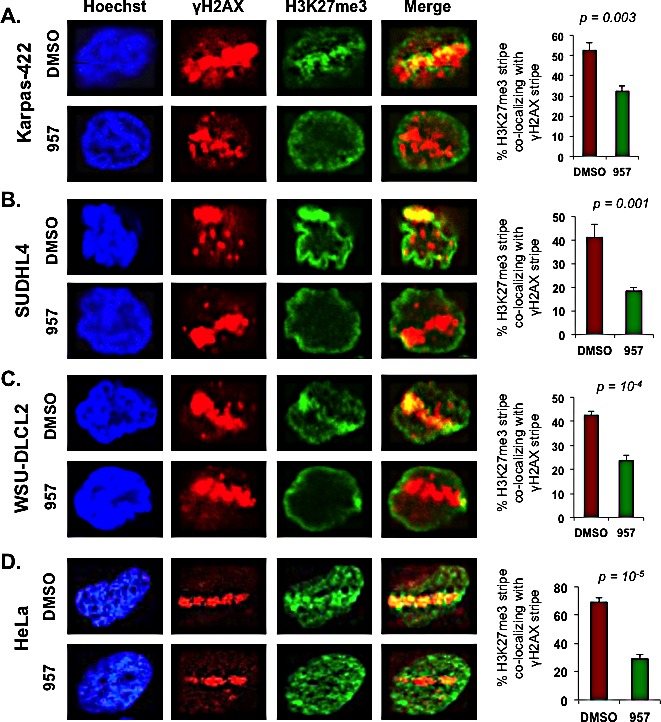
HDAC1,2 activity is critical for H3K27me3 enrichment at defined laser-induced break sites in chemoresistant DLBCL cells Karpas-422, SUDHL4, WSU-DLCL2 and HeLa cells were laser micro-irradiated and allowed to recover for 15 minutes before fixation and immunofluorescence staining with anti-γH2AX and anti-H3K27me3. Subsequently, the percentage of cells with H3K27me3 lines that co-localized with γH2AX were counted. At least 100 cells with γH2AX lines were counted in each experiment. The quantitation shown is the average calculated from independent experiments +/− standard error. Quantitation from five, seven, five and six independent experiments performed in Karpas-422, SUDHL4, WSU-DLCL2 and HeLa cells, respectively, were used in the graphs shown in this figure. Statistical analysis was performed and the p-values calculated from the t-test are shown in the figure. Merge is the overlay of γH2AX and H3K27me3 pictures.

Active recruitment of EZH2 and the subsequent active methylation of H3K27 occur during the repair process following DNA damage, as EZH2 and H3K27me3 are enriched at laser induced DNA break sites [43]. Hence, we next asked whether selective inhibition of HDAC1,2 activity impairs DNA repair in the EZH2^GOF^ DLBCL cells by affecting H3K27me3 that occurs locally at DNA break sites during repair. To address this question, we first optimized conditions to induce DNA breaks using a laser in DLBCL cells, which are smaller in size and in suspension compared to other cell types, such as U2OS or HeLa, that are adherent and typically used in the laser induced break assay. We have determined the optimal laser energy to be applied, the optimum recovery time needed to detect the recruitment of repair proteins or occurrence of histone modification at laser induced break sites in DLBCL cells and the conditions that promote the adherence of DLBCL cells onto the chamber slide (see the Methods section for details). Using the optimized assay, we have detected for the first time both γH2AX and H3K27me3 at laser-induced DNA break sites in Karpas-422 and SUDHL4 (Figure 6, see panels labeled DMSO). Elledge and colleagues showed that H3K27me3 is enriched at laser-induced break sites in HeLa cells [43], but Campbell et al reported that H3K27me3 is absent at laser-induced break sites [45]. The discrepancy between these two studies is likely to due to the difference in the time of recovery that was used prior to detecting H3K27me3. We find H3K27me3 is enriched at laser induced break sites in DLBCL cells following a 15 min recovery period following exposure to laser (Figure 6), which is consistent with the finding of Elledge and colleagues. Additionally, H3K27me3 was not enriched at laser induced break sites in DLBCL cells after a 5 min recovery, as used by Campbell et al (data not shown).

ACY-957 treatment dramatically reduced the extent of H3K27me3 present at laser-induced break sites when compared to that present at the break sites in the DMSO treated control cells (Figure 6). To confirm these results, we performed the laser-break assay in three different EZH2^GOF^ cell lines (Karpas-422, SUDHL4 and WSU-DLCL2) (Figure 6A, 6B and 6C). WSU-DLCL2 has an Y641F mutation in the SET domain [46]. We also confirmed this finding in an independent cell line (HeLa) that is routinely used in studies investigating DNA repair dynamics (Figure 6D). Similar to that observed in the refractory DLBCL cells, decreased H3K27me3 was observed at laser induced break sites in HeLa cells following ACY-957 treatment (Figure 6D). These results suggest that HDAC1,2 activity is required for EZH2-mediated H3K27me3 to occur at break sites during DNA repair. Given the enrichment of H3K27me3 along with γH2AX at the laser-induced breaks (which appear as a stripe within the nucleus, see Figure 6), we then examined whether H3K27ac forms ‘anti-stripe’ and is excluded from the laser-induced break sites. A pan-nuclear staining for H3K27ac was observed in DMSO treated control cells, which was increased following inhibition of HDAC1,2 activity using ACY-957 (Supplementary Figures 4B, 4C). H3K27ac was neither enriched at nor excluded from laser-induced break sites in DMSO or ACY-957 treated cells (Supplementary Figures 4B, 4C). These results suggest that HDAC1,2 do not remove all the H3K27ac marks at break sites; but they might target a fraction of H3K27ac (either specifically or stochastically) in order for the enrichment of H3K27me3 catalyzed by EZH2 to occur at the break sites during DNA repair. Overall, our findings suggest that selective inhibition of HDAC1,2 activity impairs DNA repair in the EZH2^GOF^ DLBCL cells in part by blocking EZH2-mediated H3K27me3.

### Chemoresistant EZH2^GOF^ DLBCL cells expressing hyperactive EZH2 also overexpress the E3 ligase BBAP

GSK126 is a highly selective and potent inhibitor of EZH2 activity, and it targets the catalytic SET domain. GSK126 treatment of SUDHL4 and Karpas-422 cells leads to a significant decrease in H3K27me3 following 48h treatment of cells with this EZH2 inhibitor (Figure 4), but viability or cell cycle progression of these chemoresistant DLBCL cells is not compromised at this time point or even after 72h post-incubation with GSK126 (Figure 3). These results suggest that a decrease in H3K27me3 alone is not sufficient to trigger death and/or cell cycle arrest in the refractory DLBCL cells in a short time frame. A prolonged 7-day incubation with GSK126 is required to decrease the viability of Karpas-422 cells [8]. EZH2^GOF^ DLBCL cells appear to be more sensitive to selective inhibition of HDAC1,2 activity than inhibition of EZH2 activity, because we observed death and/or cell cycle arrest in SUDHL4 and Karpas-422 cells as early as 48h or 72h following treatment with ACY-957 (Supplementary Figure 2 and Figure 3). These findings together suggest that in addition to H3K27me3, inhibition of HDAC1,2 activity adversely affects other factors that are required for proliferation/survival and/or chemoresistance in these refractory DLBCL cells.

**Figure 7:**
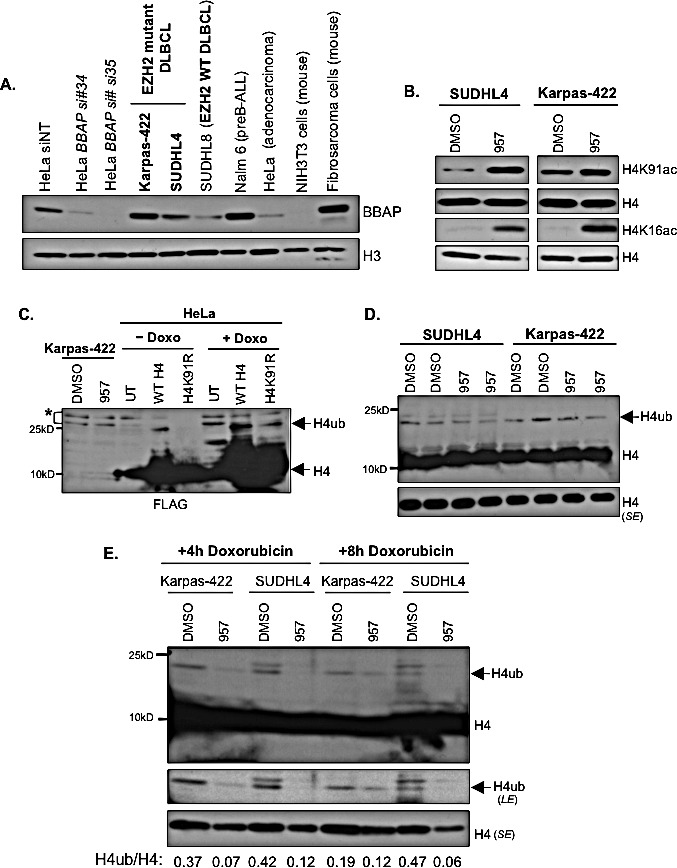
EZH2^GOF^ DLBCL cells also over express BBAP E3 ligase, HDAC1,2 target H4K91ac and inhibition of HDAC1,2 activity decreases H4 monoubiquitination following doxorubicin treatment A: Whole cell lysate was prepared from Karpas-422, SUDHL4, SUDHL8, NALM6, HeLa, NIH3T3, and mouse fibrosarcoma cell lines. Western analysis was performed with anti-BBAP antibody and histone H3 was used as a loading control. Whole cell lysate of HeLa cells was prepared following transfection of cells with either non-targeting or two different BBAP siRNAs. These extracts served as negative controls for the signal from BBAP antibody. B: SUDHL4 and Karpas-422 cells were treated with DMSO or 2μM ACY-957 for 48h prior to chromatin extraction. Western analysis was done using anti-H4K91ac or anti-H4K16ac and histone H4 served as a loading control. C: HeLa cells were transfected with either wild type H4 or H4K91R mutant constructs for 65 hours prior to extraction of histones by trichloroacetic acid. Western blot analysis was performed with anti-FLAG antibody. H4ub, ubiquitinated form of H4. * : cross-reacting bands. D: SUDHL4 cells and Karpas-422 were treated with DMSO or 2μM ACY-957 for 24h and histones were extracted by TCA precipitation. Western blotting with anti-H4 was performed to detect H4 and monoubiquitinated H4. *SE*, short exposure. E: SUDHL4 cells and Karpas-422 were treated with DMSO or 2μM ACY-957 for 48h and treated with 50nM doxorubicin for 4h or 8h prior to histone extraction. Histones were extracted by TCA precipitation and western blotting with anti-H4 was performed to detect H4 and monoubiquitinated H4. *SE*, short exposure and *LE*, long exposure.

A genetically diverse set of DLBCL cell lines was recently classified as either sensitive or resistant to chemotherapy based on responsiveness to CHOP chemotherapy regimen, which includes doxorubicin [47]. Karpas-422 is a chemoresistant DLBCL cell line and SUDHL4 DLBCL cell line is partially resistant to chemotherapy drugs when compared to the sensitive SUDHL8 DLBCL cell line [47]. Deltex (DTX)-3-like E3 ubiquitin ligase (DTX3L), also known as B-lymphoma and BAL-associated protein (BBAP), is overexpressed in high risk, chemotherapy-resistant aggressive forms of DLBCL [16]. BBAP is required for the monoubiquitination of histone H4K91 (H4K91ub1) and has been proposed to protect cells from death when exposed to DNA-damaging agents [17, 18]. Hence, we postulated that BBAP and H4K91ub1 might also contribute to the chemoresistance and/or survival of the DLBCL cells. We therefore tested whether chemoresistant EZH2^GOF^ cells also express higher levels of BBAP compared to the chemosensitive DLBCL cells. Western blotting was performed to detect BBAP levels using extracts prepared from EZH2^GOF^ lines (Karpas-422, SUDHL4), a chemosensitive EZH2 wild-type DLBCL line (SUDHL8), NALM6 (a pre-B ALL line), HeLa (a cervix adenocarcinoma line), mouse NIH3T3 cells and mouse fibrosarcoma cells. Additionally, extracts from HeLa cells transfected with either a non-targeting siRNA (siNT) or one of the two siRNAs targeting the *BBAP* transcript were included as controls. Our results showed that BBAP levels are higher in the chemoresistant Karpas-422 when compared to SUDHL4 cells and the chemosensitive SUDHL8 cells (Figure 7A). Amongst the EZH2^GOF^ DLBCL lines, Karpas-422 is more chemoresistant than SUDHL4; BBAP levels were higher in the former than the latter (Figure 7A). Additionally, BBAP levels were high in the chemoresistant NALM6 cells (Figure 7A). These results together show a correlation between chemoresistance and elevated BBAP levels. Refractory DLBCL cells contain a hyperactive EZH2, and EZH2 is important for DNA repair (Figures 5 and 6), but inhibition of EZH2 activity alone is not sufficient to cause cytotoxicity in these cells (Figure 3). BBAP is required for the efficient repair of double strand DNA breaks, and BBAP-mediated H4K91ub1 is increased upon exposure of cells to doxorubicin (a DNA-damaging chemotherapy drug) [17]. It is conceivable that increased BBAP levels in the refractory DLBCL cells might promote repair of the DNA damage induced by chemotherapy drugs, likely via increased H4K91ub1, to confer chemoresistance and support the proliferation/survival of these cells.

### Selective HDAC1,2 inhibition increases H4K91ac and decreases the DNA damage induced, BBAP-mediated ubiquitination of H4K91 in the EZH2^GOF^ DLBCL cells

The H4K91 residue is implicated in DNA repair in yeast and mammalian cells. H4K91 undergoes chemically exclusive modifications, acetylation and ubiquitination, in mammalian cells [17]. Therefore, we hypothesized that selective inhibition of HDAC1,2 activity might increase H4K91ac to block BBAP-mediated H4K91ub1 and impair DNA repair in the chemoresistant DLBCL cells. To test this possibility, we performed acid extraction of nuclei isolated from either DMSO or ACY-957 treated Karpas-422 and SUDHL4 cells to obtain histones-enriched fractions. We used the acid extraction procedure to not only enrich for the highly basic histone proteins, but to also prevent the loss of histone ubiquitination, which is a labile modification that is removed by the action of deubiquitinases within the cell. Western analysis of acid-extracted histones showed that inhibition of HDAC1,2 activity increased global H4K91ac levels in both SUDHL4 and Karpas-422 cells (Figure 7B), and therefore, revealed that H4K91ac is a novel target of HDAC1,2. Additionally, ACY-957 treatment also increased global levels of H4K16ac (Figure 7B), which we previously showed is a target of HDAC1,2 [28]. H4K16ac occurs at the N-terminal tail region and is known to disrupt chromatin packaging by preventing inter-nucleosomal interactions [48]. On the other hand, the H4K91 residue is present at the interface between the H3-H4 tetramer core and the H2A-H2B dimer within the nucleosome, and acetylation at this residue is proposed to weaken nucleosome stability by preventing the salt bridge formation and adversely affect chromatin assembly, as reported in yeast [49-51]. Therefore, our results together suggest that HDAC1,2 may play a role in nucleosome and chromatin dynamics in the chemoresistant DLBCL cells.

We next examined whether global H4K91ub1 is impacted upon selective inhibition of HDAC1,2 activity in DLBCL cells. Covalent addition of a bulky 7.6-kDa ubiquitin moiety onto the H4K91 residue retards gel migration resulting in slower migrating species of H4. We treated HeLa cells transfected with a plasmid to express a Flag-Myc epitope-tagged H4 or Flag-Myc epitope-tagged H4 mutant harboring a lysine to arginine substitution at position 91 (H4K91R) with doxorubicin. As shown in Figure 7C, H4K91ub1 induced by doxorubicin is significantly reduced by H4K91R mutation. An antibody recognizing H4K91ub1 is not available. We therefore used an anti-histone H4 antibody in the Western analysis of acid-extracted histones obtained from SUDHL4 or Karpas-422 cells treated for 30h (Figure 7D) or 48h (data not shown) with DMSO or ACY-957, which showed no change in global levels of the slow migrating monoubiquitinated form of H4. This result suggested that inhibition of HDAC1,2 activity does not affect preexisting H4K91ub1 marks. H4K91ub1 levels are increased when cells are exposed to DNA-damaging agents, such as, doxorubicin [17]. Given the increase in global H4K91ac with ACY-957 treatment (Figure 7B), we next asked whether active monoubiquitination of H4K91 catalyzed by BBAP is regulated by HDAC1,2 activity. We treated Karpas-422 or SUDHL4 cells with DMSO or ACY-957 for 48h prior to the incubation for 4h or 8h in the presence of doxorubicin. Western analysis of acid-extracted histones showed a significant reduction to complete absence of the slow migrating monoubiquitinated form of H4 in ACY-957-treated Karpas-422 or SUDHL4 cells, respectively (Figure 7E). Collectively, these results showed that HDAC1,2 activity are required for BBAP-catalyzed active H4K91ub1 to occur when DLBCL cells are exposed to doxorubicin, a chemotherapy drug and a DNA-damaging agent.

### Selective inhibition of HDAC1,2 activity delays the kinetics of BBAP-dependent 53BP1 recruitment to chemo-induced DNA break sites

Knockdown of BBAP delayed the accumulation of 53BP1 (a key repair protein) at DNA breaks induced by doxorubicin, suggesting that BBAP, via H4K91ub1, might protect DLBCL cells from DNA damage by facilitating 53BP1-mediated repair of double-strand DNA breaks [17]. Active BBAP-mediated H4K91ub1 induced by doxorubicin is inhibited when cells are pretreated with ACY-957 (Figure 7). Therefore, we hypothesized that selective inhibition of HDAC1,2 activity might impair DNA repair in chemoresistant EZH2^GOF^ DLBCL cells by delaying the recruitment of 53BP1 to DNA break sites as a result of increased H4K91ac and decreased H4K91ub1.

**Figure 8:**
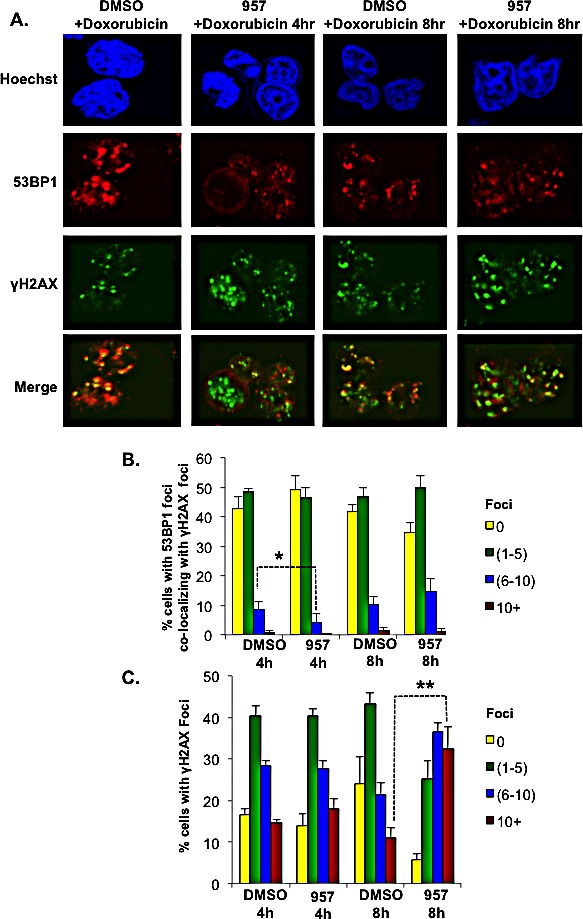
HDAC1,2 inhibition delays the kinetics of 53BP1 foci formation in refractory EZH2^GOF^ DLBCL cells A: Karpas-422 cells were treated with either DMSO or 2μM ACY-957 for 24h before the addition of doxorubicin. After 4h or 8h of doxorubicin treatment, cells were fixed and immunofluorescence staining was performed with anti-γH2AX and anti-53BP1 antibodies. Cells shown are representative images based on the quantitation. B and C: Graphs depict the percentage of cells with 53BP1 foci present at γH2AX containing break sites and the percentages of cells with γH2AX foci. The data represents the average +/− standard error from four independent experiments. *p=0.05; **p=0.009. Merge is the overlay of γH2AX and 53BP1 pictures.

To test this possibility, we treated DLBCL cells with DMSO or ACY-957 for 24h prior to a low dose of doxorubicin (50nM) treatment. We examined γH2AX and 53BP1 foci formation at 4h or 8h post-doxorubicin treatment using immunofluorescence. We chose a 24h ACY-957 treatment for this assay as we did not detect any significant accumulation of endogenous DNA damage resulting from inhibiting HDAC1,2 activity using this treatment time (data not shown), unlike that observed when cells were treated with ACY-957 for 48h (Figure 5). Thus, this short duration of inhibitor treatment enabled us to bypass the DNA repair events triggered by endogenous DNA damage in the absence of HDAC1,2 activity and directly follow the kinetics of doxorubicin-mediated DNA repair upon inhibiting HDAC1,2. Immunofluorescence showed co-localization of 53BP1 with γH2AX at 4h and 8h following addition of doxorubicin to the control DMSO-treated Karpas-422 cells (Figure 8).

The percentage of cells containing 0, 1-5, 6-10 and greater than 10 γH2AX and 53BP1 foci were determined in at least 100 cells from four independent experiments. Quantitation of percentage of cells with 53BP1 foci co-localizing with γH2AX foci revealed a decrease in the number of 53BP1 foci in ACY-957-treated Karpas-422 cells at 4h post-doxorubicin treatment when compared to DMSO-treated controls (Figure 8). Following an 8h treatment with doxorubicin, the percentage of cells with 1-5, 6-10, and greater than 10 53BP1 foci in ACY-957-treated cells increased to the level that was observed in the control DMSO-treated cells. However, ACY-957-treated cells had an even higher percentage of cells with 6-10 and greater than 10 γH2AX foci compared to DMSO-treated control cells at this 8h time point (Figure 8). These results together suggest that HDAC1,2 activity are required for the initial recruitment of 53BP1 to DNA break sites following exposure to doxorubicin. Our results also suggest that pretreatment of EZH2^GOF^ DLBCL cells with HDAC1,2-selective inhibitor sensitizes cells to doxorubicin which results in increased DNA damage response and increased number of γH2AX-containing double-strand breaks. However, the repair of these breaks is impaired in the absence of HDAC1,2 activity as the majority of γH2AX-containing break sites do not also contain 53BP1 repair protein.

### Treatment with HDAC1,2 selective inhibitor sensitizes the EZH2^GOF^ refractory DLBCL cells to doxorubicin

**Figure 9:**
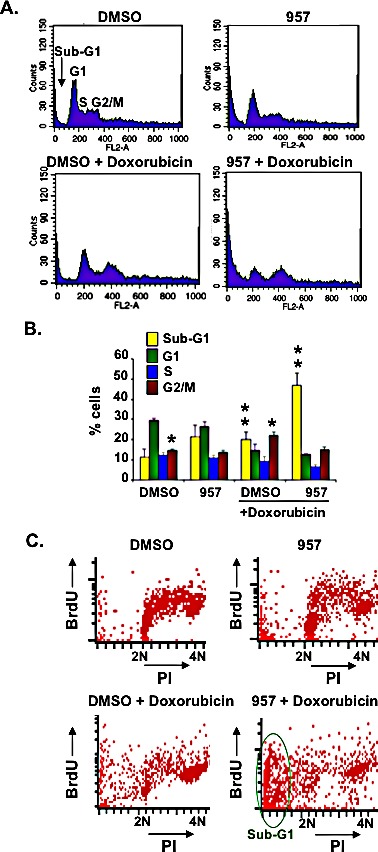
HDAC1,2 inhibition sensitizes chemoresistant Karpas-422 cells to doxorubicin induced cell death A: Karpas-422 cells were treated with DMSO, 2μM ACY-957, 50 nM doxorubicin or ACY-957 plus 50nM doxorubicin for 48h. Cell cycle analysis of propidium-iodide stained cells was performed with fixed cells. Representative plots from five independent experiments are shown in the figure. B: The graph represents average percentage cells +/− standard error of five independent experiments. *p= 0.003, **p= 0.005. C: FACS analysis following BrdU-PI staining of Karpas-422 cells was performed following a treatment with 2μM ACY-957, 50nM doxorubicin or both for 48h. Representative plots from two independent experiments are shown in the figure.

BBAP knockdown sensitizes HeLa cells to doxorubicin-induced cell death [18] and inhibition of HDAC1,2 activity decreased H4 monoubiquitination and 53BP1 recruitment during doxorubicin-induced DSB repair (Figures 7 and 8). Hence, we examined whether HDAC1,2 inhibition could sensitize the chemorefractory EZH2^GOF^ Karpas-422 cells to doxorubicin treatment and overcome drug resistance. In order to test this possibility, we performed cell cycle analysis following treatment of Karpas-422 with ACY-957, doxorubicin or ACY-957 in combination with doxorubicin. Karpas-422 cells showed a 1.5 to 2-fold increase in the percentage of cells in G2/M phase with a modest increase in cell death upon doxorubicin treatment (Figures 9A, 9B). Additionally, a sub-G1 population (i.e., dead cells) was observed following ACY-957 treatment of Karpas-422 cells (Figures 9A, 9B). However, combined treatment of Karpas-422 cells with doxorubicin and ACY-957 resulted in an increased sensitivity of cells to death, as the sub-G1 population increased by ~5-fold when compared to DMSO control (Figures 9A, 9B). BrdU-PI analysis also showed a consistent increase in sub-G1 population upon combined application of ACY-957 and doxorubicin to Karpas-422 cells (Figure 9C). Our results therefore suggest that HDAC1,2 inhibition can overcome doxorubicin-resistance in the refractory EZH2^GOF^ Karpas-422 cells by impairing BBAP-mediated DSB repair via H4K91ub1 in addition to EZH2-mediated DSB repair via H3K27me3.

## DISCUSSION

### HDAC1,2-selective inhibition acts on the H3K27ac−H3K27me3 balance in the EZH2^GOF^ DLBCL cells

Approximately 20% of germinal center-derived DLBCL cells have an activating mutation in the SET domain of EZH2 that catalyzes aberrantly high H3K27me3 [7]. Addition of GSK126, a potent and selective small molecule inhibitor of EZH2 activity, decreases H3K27me3, reactivates PRC2-suppressed genes, such as, *BLIMP1* (a tumor suppressor) and induces cytotoxicity in these lymphoma cells [8]. These findings implicate a role for the hyperactive mutant EZH2 enzyme and the aberrantly increased H3K27me3 to the growth and survival of these GC-DLBCL cells. As a complement to the studies using GSK126, we show that selective inhibition of HDAC1,2 activity also causes cytotoxicity or cell cycle arrest in the EZH2^GOF^ GC-DLBCL cells. However, unlike GSK126, inhibition of HDAC1,2 does not decrease the aberrantly increased global H3K27me3, but instead leads to a robust increase in global H3K27ac in these GC-DLBCL cells (Figure 4). In the absence of DNA damage, the promoters of DNA damage response genes are repressed and thus, expected to have high H3K27me3, a mark generally associated with inactive genes. Since acetylation and methylation are chemically exclusive modifications that occur on the ε-amino group of lysine residues, removal of methylation is required prior to acetylation and vice versa. Upon inhibition of HDAC1,2 activity and the ensuing transcriptional activation, H3K27ac is increased at the promoters of the DNA damage response genes without a concomitant decrease in H3K27me3. Therefore, our results demonstrate that demethylation or removal of preexisting H3K27me3 marks, either globally or locally, is not necessary for the increase in chromatin-associated H3K27ac to occur. How then can acetylation at H3K27 occur without the removal of H3K27me3? Since each nucleosome contains two H3 subunits, acetylation can occur on the unmodified H3K27 present within the same nucleosome as the trimethylated H3K27 or alternatively, acetylation might occur on the unmodified H3K27 present in the neighboring nucleosome. Therefore, one can envision the presence of an interesting and intricate mechanism underlying the lymphomagenesis and survival of the EZH2^GOF^ DLBCL cells involving the maintenance of the fine balance between acetylation and methylation at the H3K27 residue. In the DLBCL cells, HDAC1,2 via their interaction with PRC2 containing the hyperactive EZH2 maintain an aberrant ‘H3K27me3>H3K27ac’ state, which likely promotes the lymphomagenesis and/or survival of these lymphoma cells, through repressing the DNA damage response genes. Inhibiting EZH2 activity or HDAC1,2 activity therefore shifts the balance towards the ‘H3K27ac>H3K27me3’ state, which might lead to the activation of select DNA damage response genes and trigger either cell cycle arrest or death in the EZH2^GOF^ mutant DLBCL cells.

### Regulation of EZH2-dependent DSB repair by HDAC1,2

HDAC1,2 localize to double-strand break (DSB) sites and play a role during DSB repair [44, 52]. Cancer cells are in general ‘addicted’ to DNA repair and use DSB repair as one survival mechanism to overcome cytotoxic effects of chemotherapeutic agents. Polycomb group proteins have been extensively studied in the context of transcription. Linking these proteins to genome maintenance, increased association of polycomb proteins with chromatin was observed in a screen for chromatin-associated proteins following DNA damage [43]. The H3K27 methyltransferase EZH2 is the catalytic component of polycomb group PRC2 complex. Depletion of EZH2 by shRNA makes cells sensitive to IR treatment [45], suggesting a role for EZH2 in DSB repair. EZH2 localizes to DSB sites and catalyzes H3K27me3, and both these factors are enriched at DSB sites following DNA damage [43]. Hence, increased H3K27me3 mediated DSB repair might be one mechanism by which EZH2^GOF^ DLBCL cells might develop resistance to chemotherapeutic agents. We show that inhibition of HDAC1,2 activity decreases H3K27me3 at break sites and activates the DNA damage response in the EZH2^GOF^ DLBCL cells (Figures 5 and 6). Hence, HDAC1,2 inhibition overcomes H3K27me3-mediated DSB repair and sensitizes the EZH2^GOF^ DLBCL cells to DNA damage. Even though H3K27me3 is enriched at laser-induced break sites, H3K27ac is not excluded from the break sites, as it does not form ‘anti-stripe’ (Supplementary Figure 4). How do HDAC1,2 then regulate H3K27me3 at break sites? One possibility is that HDAC1,2 might target an acetyl mark other than H3K27ac that regulates EZH2-mediated H3K27me3. Alternatively, we favor a model where HDAC1,2 might remove only a few H3K27ac marks localized around break site which is sufficient to promote the addition of new H3K27me3 marks, but insufficient to be detected by immunofluorescence technique.

H3K27me3 is a transcriptional repression mark [23]. What might the function of a transcriptional repression histone mark be at DSB sites? Transcription by RNA polymerase II is transiently repressed at break sites to prevent the transcription machinery from interfering with the ongoing repair process [53-55]. Also, transcriptional silencing might prevent any destabilizing changes in the chromatin around break sites by establishing a more condensed, heterochromatin-like state that maintains the broken chromosomal ends in close proximity to each other for efficient repair. Inhibition of HDAC1,2 activity via the effect on histone acetylation and chromatin may relieve this transcriptional repression at damage sites to impair the ongoing DNA repair. In addition to polycomb complexes (PRC1 and PRC2), the NuRD complex also promotes transcriptional repression at break sites [43]. HDAC1,2 are the catalytic components of the NuRD complex. Therefore, HDAC1,2 inhibition would not only impair polycomb-mediated transcription at break sites but could also impair NuRD-mediated transcriptional repression at DSB sites.

Apart from maintaining transcriptional repression at break sites, H3K27me3 also acts as a docking site for a second polycomb complex PRC1 during DSB repair signaling [56]. PRC1 maintains gene repression via ubiquitination of H2AK119 [56]. H2AK119 ubiquitination mediated by the PRC1 complex helps in the recruitment of several repair factors, such as, BRCA1 and 53BP1 and perpetuates the damage signaling [57]. Hence, reduced accumulation of H3K27me3 at break sites in the absence of HDAC1,2 could also impair the recruitment of PRC1 complex and downstream EZH2-mediated H2AK119 repair signaling, the hypothesis that is currently under investigation.

### Regulation of BBAP-mediated DSB repair by HDAC1,2

In a screen to identify genes associated with the curable versus fatal DLBCLs, a gene called BAL (B-aggressive lymphoma) was highly expressed in fatal tumors [58]. A yeast two-hybrid screen identified BBAP or DTX3L as a BAL-binding protein [15]. BBAP contains a C-terminal RING-finger domain that monoubiquitinates H4K91 [15, 17]. BBAP enzyme is most abundant in DLBCLs with a prominent immune infiltrate and increased IFNγ production [16]. BBAP monoubiquitinates H4K91, promotes the recruitment of methyltransferase for H4K20 methylation, aids the DNA repair process and protects cells from DNA damaging agents to promote chemoresistance [17]. Inhibition of HDAC1,2 activity leads to a more rapid cell death compared to inhibition of EZH2 activity alone, suggesting that factors in addition to H3K27me3 might contribute to the chemoresistance in EZH2^GOF^ DLBCL. This possibility prompted us to examine BBAP levels in the chemoresistant EZH2^GOF^ DLBCL cells. We find that the refractory EZH2^GOF^ DLBCL cells expressing a hyperactive EZH2 enzyme also overexpress the enzyme BBAP. Hence, the BBAP activity likely contributes to chemoresistance in addition to the EZH2 activity, and thus accounts for the difference in the efficacy of GSK126 and ACY-957 on the EZH2^GOF^ DLBCL cells. This may also explain why refractory EZH2^GOF^ DLBCL cells, such as Karpas-422, respond slowly to EZH2 inhibition compared to HDAC1,2 inhibition (7 days for GSK126 versus 3 days for ACY-957) [8], as BBAP activity in these DLBCL cells might protect these cells from any DNA damage even in the absence of EZH2 activity.

Inhibition of HDAC1,2 activity in the EZH2^GOF^ DLBCL cells increases acetylation at H4K91, the residue monoubiquitinated by BBAP (Figure 7). H4K91ubiquitination is required for efficient 53BP1 recruitment to damage sites at early time points during repair [17]. We observed a delay in the kinetics of 53BP1 foci formation upon HDAC1,2 inhibition (Figure 8) similar to that observed in *BBAP* knockdown cells [17]. The H4K91 ubiquitination induced by DNA damage is abolished upon HDAC1,2 inhibition. Therefore, HDAC1,2 inhibition overcomes BBAP-mediated chemoresistance by increasing H4K91ac to inhibit H4K91 ubiquitination and thus, impair DSB repair mediated by 53BP1 recruitment to triggering DNA damage in the lymphoma cells. Recently, an increase in H4K16ac was reported to interfere with the H4K20me-binding by 53BP1 and its localization to break sites [59]. In addition to H4K91ac, an increase in H4K16ac was also observed in ACY-957-treated EZH2^GOF^ DLBCL cells (Figure 7B). Hence, the increase in H4K16ac is another mechanism by which 53BP1 recruitment to break sites is impaired by inhibition of HDAC1,2. H4K91ac is also involved in chromatin assembly during DNA repair in yeast. H4K91ac is proposed to influence nucleosome assembly by regulating interactions between the H3-H4 tetramer core and the H2A-H2B dimers [50]. During DNA damage response and repair, proper chromatin reassembly is essential to maintain genome stability. Hence, investigation of the mechanism by which HDAC1,2 modulate chromatin assembly following DNA repair via H4K91 deacetylation is warranted in future studies.

### A novel HDAC1,2-targeted therapy for refractory EZH2^GOF^ DLBCL

**Figure 10:**
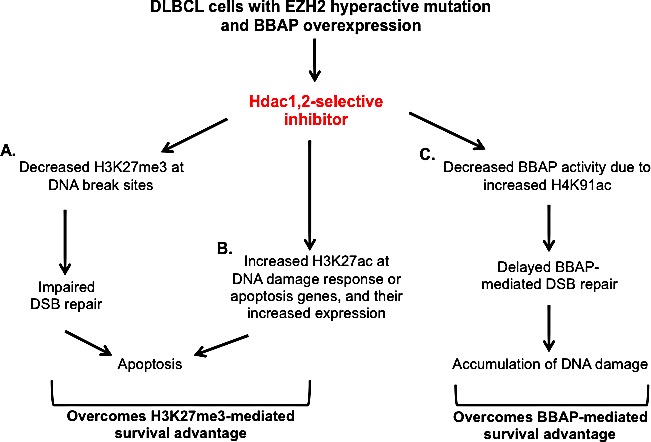
Model for the mechanism of action of HDAC1,2-selective inhibitor in EZH2^GOF^ DLBCL cells A: HDAC1,2 selective inhibition decreases H3K27me3 at break sites to impair DSB repair and activate DNA damage response. B: HDAC1,2 inhibition increases H3K27ac at DNA damage response genes to increase their transcription. A and B together overcomes the survival advantage provided by increased H3K27me3 in these EZH2^GOF^ DLBCL cells. C: HDAC1,2 inhibition increases H4K91ac and decreases H4K91 monoubiquitination following doxorubicin treatment. This results in a defective 53BP1 recruitment to damage sites and defective repair of doxorubicin-induced breaks. Hence, HDAC1,2 inhibition overcomes BBAP-mediated chemoresistance following doxorubicin treatment in DLBCL cells with EZH2 hyperactive mutation.

A major advancement in the treatment of DLBCL has been made with the addition of anti-CD20 monoclonal antibody (rituximab) to the standard chemotherapy regimen (CHOP) [2, 60]. However, a significant number of patients (~40%) have treatment failure and relapse after the initial response to R-CHOP treatment and no regimen has been proven to be superior with regard to the overall survival of DLBCL patients [2, 60]. Hence there is an urgent need for novel therapeutics to treat relapsed patients. Overall, in this study, we have made connections between three important chromatin-modifying enzymes (HDAC1,2, EZH2 and BBAP) that coordinately play a role in DNA repair to modulate chemoresistance in the EZH2^GOF^ DLBCL cells. We show that inhibition of HDAC1,2 is sufficient to overcome the survival advantage mediated by EZH2 and BBAP enzymes in the refractory EZH2^GOF^ DLBCL cells (Figure 10). HDAC1,2-selective inhibitor interferes or overcomes the activity of EZH2 and BBAP at the H3K27 and H4K91 residues, respectively, to potentially inhibit DSB repair at various steps: DNA damage signaling mediated by BBAP, transcriptional repression at break sites mediated by EZH2 and chromatin changes around break sites during DNA repair mediated by H4K91ac. In addition to impairing DSB repair, we find activation of DNA damage response genes, such as, *BMF* and *Tp63* upon HDAC1,2 inhibition. Previously, the molecular responses of isogenic normal and transformed cells to the pan-HDAC inhibitors, Vorinostat and Romidepsin, were examined [61]. Both these pan-HDIs selectively killed transformed cells and induced tumor cell-selective up regulation of the pro-apoptotic *BMF* gene [61]. We find that selective HDAC1,2 inhibition alone is sufficient to increase *BMF* gene transcription in the refractory EZH2^GOF^ DLBCL cells, and therefore, pan-HDI induction of *BMF* gene expression is likely to be mediated in large part due to their inhibition of HDAC1,2 activity. Hence, HDAC1,2 inhibition appears to overcome chemoresistance mediated by both EZH2 and BBAP by affecting regulators of genome stability in the chemoresistant EZH2^GOF^ DLBCL cells and by activating the transcription of DNA damage response genes independent of EZH2 function. Therefore, our results show that the selective inhibition of HDAC1,2 activity is a promising DNA repair mechanism-based therapeutic approach for chemoresistant EZH2^GOF^ DLBCL. Examination of HDAC1,2 selective inhibitors in clinical trials is warranted, in combination with doxorubicin or other therapeutics with the potential advantage that chemoresistance in the refractory EZH2^GOF^ DLBCL may be mitigated to trigger increased cell death. Currently, inhibiting DNA repair mechanisms using a HDAC1,2-selective inhibitor as a therapeutic strategy for other cancers is under investigation.

## MATERIALS AND METHODS

### Cell Culture and Chemicals

HeLa cells were cultured in DMEM containing 10% fetal bovine serum (Hyclone, Logan, UT, USA), 1% penicillin-streptomycin and 1% glutamine. Karpas, SUDHL4 and SUDHL8 cells were cultured in RPMI supplemented with 20% heat inactivated fetal bovine serum (Hyclone, Logan, UT, USA), 1% penicillin-streptomycin, 1% L-glutamine, 0.002% HEPES, 0.1% amphotericin B. NIH3T3 cells were cultured in DMEM (Cellgro™, Tewksbury, MA, USA) containing 10% fetal calf serum, 1% penicillin-streptomycin and 1% glutamine. *Hdac1,2* and *Hdac3* conditional knockout fibrosarcoma cells were cultured and infected with Ad-Cre as described previously [29]. DZNep was purchased from Cayman Chemicals and GSK126 was purchased from Xcess Bio. BBAP siRNAs were purchased from Qiagen. A lentiviral plasmid construct containing the histone H4 cDNA along with myc and Flag epitope tags was purchased from Origene. Standard PCR-based mutagenesis was used to introduce the H4K91R mutation into this plasmid. The introduced mutation in histone H4 was confirmed by sequencing.

### Antibodies

The following antibodies were purchased from Abcam: H4, H4K5ac, H4K91ac, BBAP, γH2AX for western blot, H4K16ac. H3K23ac, H4K16ac, H2AX and H3K27ac antibodies were purchased form Active Motif. 53BP1 antibody was purchased from Bethyl Laboratories. Anti-H3K27me3 and EZH2 were purchased from Cell Signaling. γH2AX for immunofluorescence, H3K9K14ac and H3 antibodies were purchased from Millipore.

### Laser Micro-irradiation repair assay in HeLa cells

HeLa cells were seeded in 8-well LabTek II chamber dishes. After three hours, the media was removed and replaced with fresh media containing HDAC inhibitors in the described concentrations. After 24 hours of drug treatment, cells were pre-sensitized with 1 μg/ml Hoechst 33342 for ten minutes. Laser microirradiation was performed on an inverted confocal microscope (A1R Confocal System, Nikon), using a 405 nm laser focused through a 60x oil objective. Laser output was set to 100%, with twelve regions of interest (ROIs) at a scan speed of 1/16 and pixel dwell of 56.7, which was sufficient to produce detectable damage without noticeable cytotoxicity. Microirradiation was performed along parallel lines (ROIs) that spanned each field of view. Cells were allowed to recover for 15 minutes, at which time they were washed once with PBS and fixed for ten minutes in 10% formalin, followed by immunofluorescence staining after permeabilization with 0.5% Triton-X for 4 minutes.

### Optimization of laser irradiation repair assay in suspension B cells

To perform the laser irradiation protocol in B cells, we optimized the utilization of Cell-Tak (Corning Inc.) reagent to allow cell adherence. One hour before microirradiation, the 8-well LabTek II chamber dish was treated with the Cell-Tak solution for 30 minutes. Cell-Tak, 0.1 M, pH 8.0 sodium bicarbonate, and 1M sodium hydroxide were combined to a volume of 100μl/cm^2^ per well (2.5μl Cell-Tak, 1.25 μl sodium hydroxide and 96.25μl sodium bicarbonate). After 30 minutes, the solution was removed and the wells were washed with filter-sterilized water. HDAC inhibitor treated cells were then added to wells and allowed to settle for 30 minutes, with Hoechst 33342 being added 20 minutes into this incubation. DLBCL cells used in these experiments were treated with HDAC inhibitor for 24 hours prior in 6-well dishes. Laser output was set to 100%, with twenty-four ROIs, a scan speed of 1/24 and pixel dwell of 86.3. The autofocus ability (Perfect Focus System) of the microscope was also utilized in order to keep the smaller DLBCL cells in focus for the laser. A special stage was created to hold the 8-well chamber dishes and to keep the cells in focus. Recovery and fixation were the same as in HeLa cells, but permeabilization during immunofluorescence staining was modified. Permeabilization with either 0.1% Triton-X for 4 minutes or with ice-cold acetone for 10 minutes at −20°C was used for DLBCL cells. Images were taken with a Nikon A1R confocal microscope. Imaging was controlled by a pixel dwell of 5.1 at a size of 2048 pixels. The 405/488/561 dichroic mirror and 60x oil lens were used for all imaging.

### Histone Extraction by Trichloroacetic Acid

DLBCL cells were treated with ACY-957 in 25cm^2^ flasks. Following treatment, cells were pelleted, washed with PBS and the pellet was re-suspended in 400μl of lysis buffer with protease inhibitors (200μl 0.5M HEPES.KOH, pH 7.9, 15μl 1M MgCl_2_, 50μl 2M KCl, 50μl 0.1M DTT, 100μl 0.4M NEM, 10μl 1000x Aprotinin/Leupeptin, 10μl 1000x Pepstatin A, and water to a total volume of 10mL plus Roche protease inhibitor cocktail). To the cell suspension, a final concentration of 0.2M sulfuric acid was added and samples were sonicated twice using the Fisher FB120 sonicator at amplitude of 50% and with 5 pulses. After sonication, proteins were extracted for 30 minutes at 4°C with end over end rotation. Samples were then centrifuged at 13,000 rpm for 10 minutes at 4°C and histones were precipitated with a 20% final concentration of trichloroacetic acid and incubated on ice for 30 minutes or moved directly to −80°C overnight. Following precipitation, samples were centrifuged again for 10 minutes and the resulting pellet was washed with cold acidified acetone (acetone+0.05N HCl). The histone pellets were centrifuged at the same settings but for 5 minutes then washed again with cold acetone. After this wash the pellets were centrifuged for another 5 minutes and the acetone was discarded. Pellets were dried in an incubator at 37°C for 5 minutes then re-suspended in 50μl 2x SDS sample buffer+β-mercaptoethanol and 8 μl 1M Tris HCl pH 8 to neutralize. The samples were then boiled for 8 minutes at 95°C and if there was still visible pellet they were either boiled longer, sonicated, had more sample buffer added or a combination thereof.

### Chromatin Immunoprecipitation Assay

ChIP assays were performed as described previously [29]. Primer sequence for *TP63* and *BMF* promoters are available upon request.

### RNA-Seq Analysis

Total RNA was isolated from Karpas-422 cells that were treated with DMSO, 2μM ACY-957 for 24 h using the Versagene RNA isolation kit (5 Prime). The treatments were done in triplicate and sequenced using the Illumina Hiseq2000 sequencer. Illumina TruSeq Stranded sequencing following RiboZero treatment was performed.

### Immunofluorescence

Cells were fixed in either 10% formalin or methanol-acetone (1:1) for 10 min at −20°C. The cells were then permeabilized with 0.5% Triton-X-100 in PBS for 5 min at room temperature (RT), blocked in 10% normal goat serum (Sigma) for 30 min and stained with primary antibody for 1 h at RT. Cells were incubated with secondary antibody (anti-mouse or anti-rabbit IgG, Alexa Fluor 488/546) at a 1:600 dilution for 45 min at RT, and counterstained with Hoechst 33342 (Sigma) at 1:500 dilution to visualize the nuclei. Images were captured using Zeiss Axioskop mot plus microscope and analyzed using the AxioVision software.

### Propidium-iodide cell cycle analysis

Propidium-iodide cell cycle analysis was performed as described previously [29].

### BrdU-PI cell cycle analysis

BrdU-PI cycle analysis was performed as described previously [29].

### Chromatin Extraction

Chromatin extracts for western blot analysis were prepared as described previously [28].

## SUPPLEMENTARY MATERIAL FIGURES


