# Supplementary material for: HDAC1,2 inhibition impairs EZH2- and BBAP- mediated DNA repair to overcome chemoresistance in EZH2 gain-of-function mutant diffuse large B-cell lymphoma
Source: Oncotarget. 2014 Dec 31;6(7):4863–87. doi: 10.18632/oncotarget.3120 (PMC4467121; doi:10.18632/oncotarget.3120)
Supplement: Supplementary file 1 [file oncotarget-06-4863-s001.pdf]

HDAC1,2 inhibition impairs EZH2- and BBAP- mediated DNA repair to overcome chemoresistance in EZH2 gain-of-function mutant diffuse large B-cell lymphoma

Supplementary Material

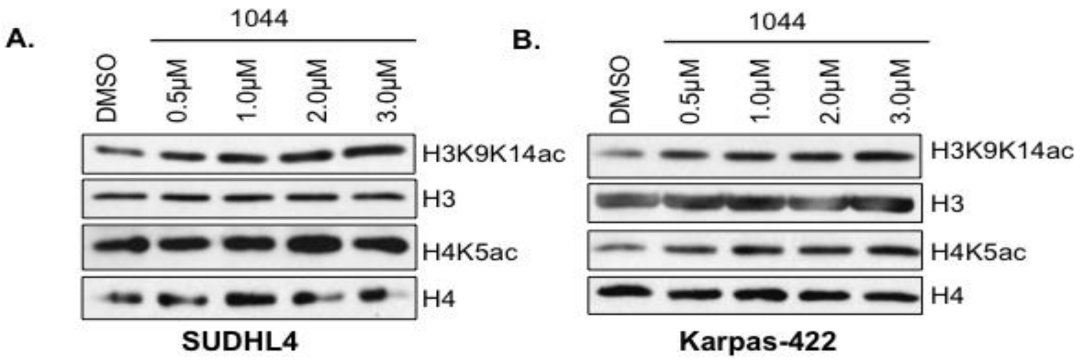

Supplementary Figure 1: SUDHL4 (**A**) and Karpas-422 (**B**) cells were treated with increasing concentrations of ACY1044 and whole cell lysates were prepared following a 24h treatment. Western blot analysis of H3K9K14ac and H4K5ac was performed. Histone H3 and H4 were used as controls.

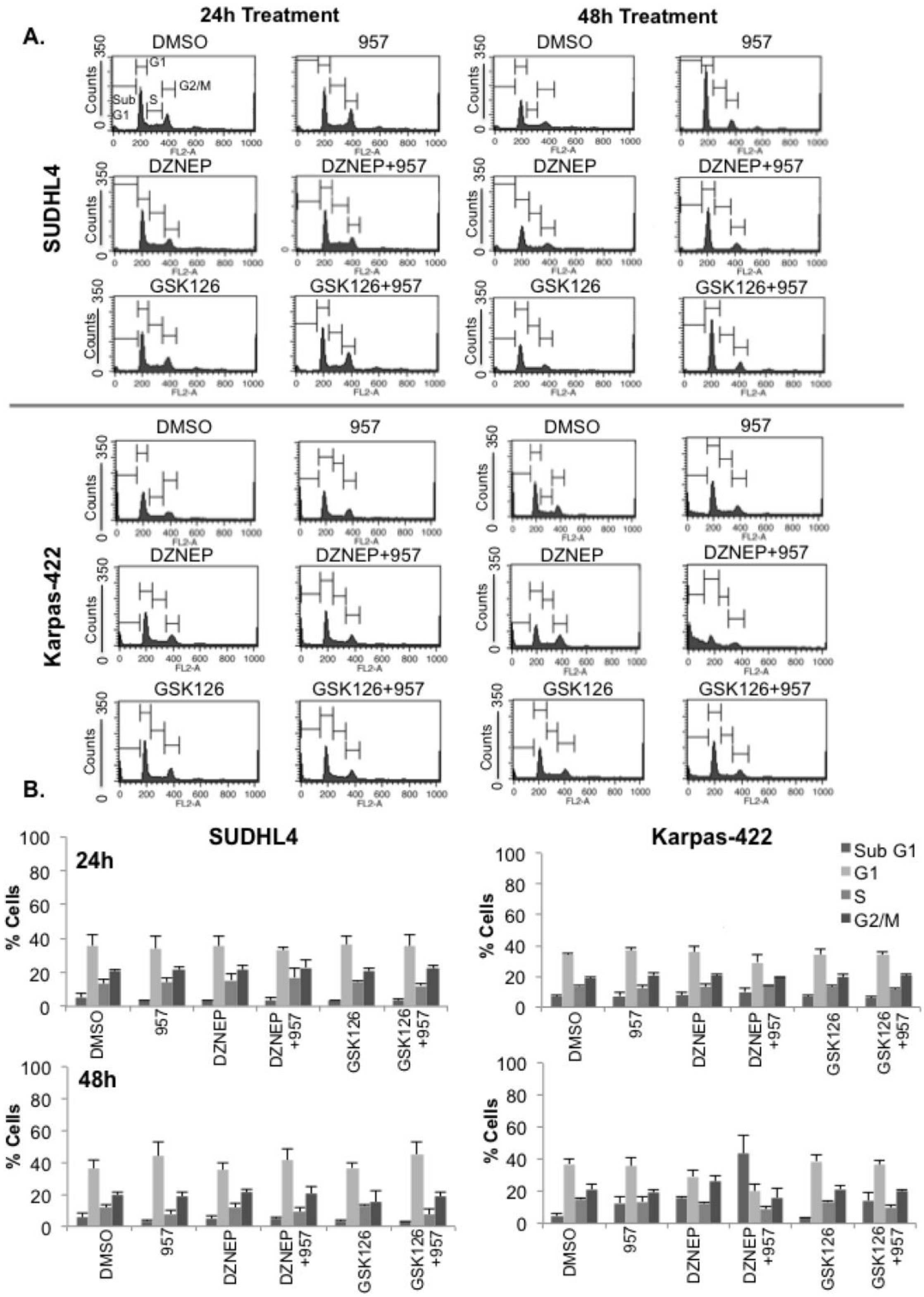

24h Treatment
48h Treatment
A.
SUDHL4
Karpas-422
DMSO
957
DZNEP
DZNEP+957
GSK126
GSK126+957
Counts
FL2-A
Sub G1
G1
S
G2/M
B.
SUDHL4
Karpas-422
24h
48h
% Cells
Sub G1
G1
S
G2/M

Supplementary Figure 2: A: SUDHL4 and Karpas-422 were treated with DMSO, 2μM ACY-957, 0.5μM DZNep, 0.5μM GSK or a combination of drugs for 24 or 48 h and cell cycle analysis of propidium-iodide stained cells was performed. Representative plots are shown in the figure. **B:** Quantitation of the cell cycle analysis performed in **A**. Data from three independent cell cycle analyses were compiled and average percentage cells with standard errors are shown in this panel.

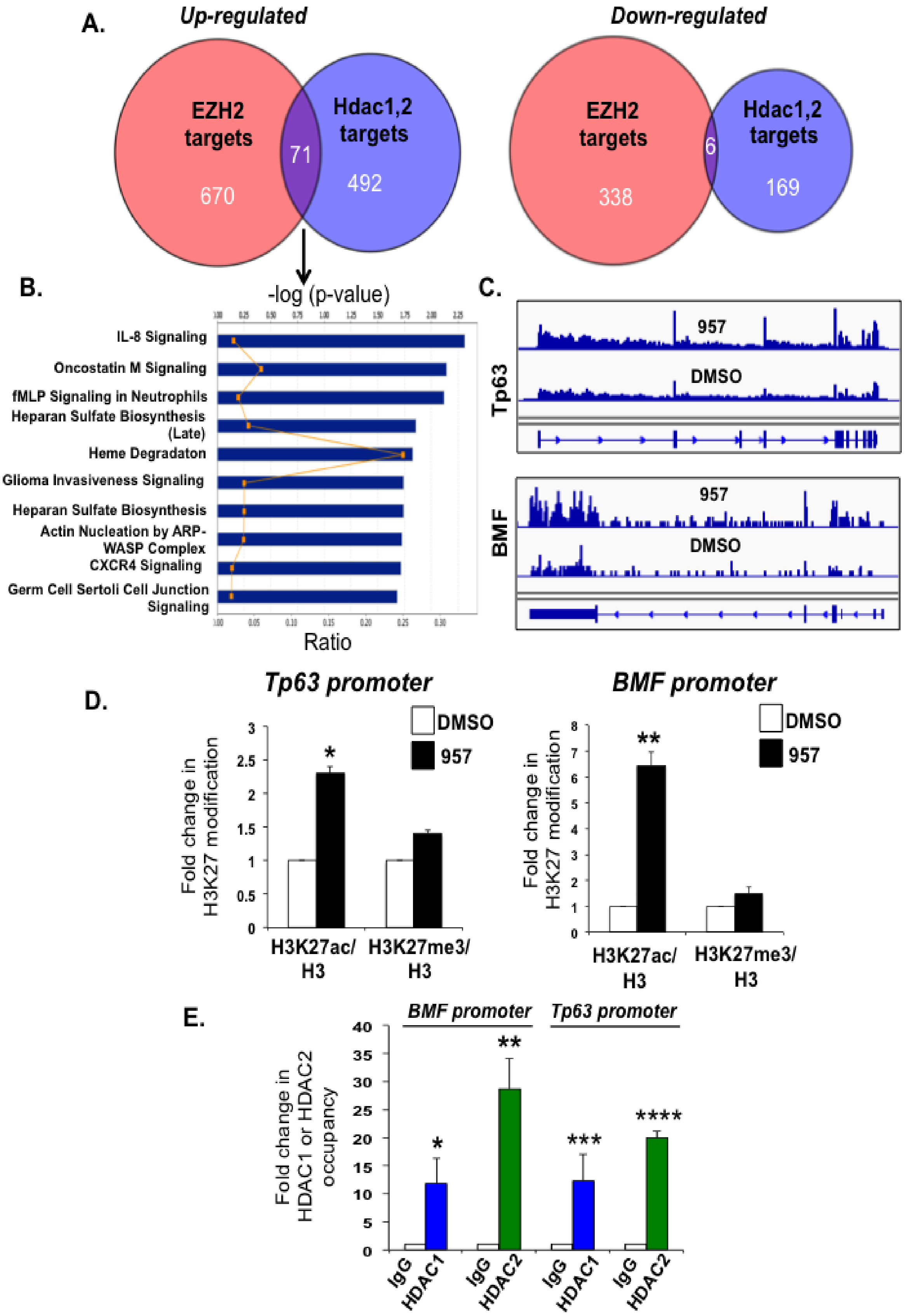
A.
Up-regulated
EZH2 targets
670
71
Hdac1,2 targets
492
Down-regulated
EZH2 targets
338
6
Hdac1,2 targets
169
B.
-log (p-value)
IL-8 Signaling
Oncostatin M Signaling
fMLP Signaling in Neutrophils
Heparan Sulfate Biosynthesis (Late)
Heme Degradaton
Glioma Invasiveness Signaling
Heparan Sulfate Biosynthesis
Actin Nucleation by ARP-WASP Complex
CXCR4 Signaling
Germ Cell Sertoli Cell Junction Signaling
Ratio
C.
Tp63
957
DMSO
BMF
957
DMSO
D.
Tp63 promoter
BMF promoter
Fold change in H3K27 modification
DMSO
957
H3K27ac/ H3
H3K27me3/ H3
E.
BMF promoter
Tp63 promoter
Fold change in HDAC1 or HDAC2 occupancy
IgG
HDAC1
IgG
HDAC2
IgG
HDAC1
IgG
HDAC2

Supplementary Figure 3: A: Total RNA was isolated from Karpas-422 cells following a 24h treatment with 2μM ACY-957 for 24h. RNA samples obtained from three independent treatments were subjected to RiboZero stranded sequencing using the Illumina Hiseq2000 sequencer. RNA-seq data from ACY-957-treated samples was compared to the publically available RNA seq data set from Karpas-422 cells following GSK126 treatment of Karpas-422 cells [38]. Venn diagrams were made using the R package VennDiagram. Genes with adjusted p-value < 0.05 and absolute log2 fold change > 0.585 were used in this analysis. **B:** Pathway analysis of the 71 commonly up regulated genes was performed. Screen shots of the pathways that are misregulated in both ACY-957-treated Karpas-422 cells and in Karpas-422 cells following GSK-126 treatment [38]. Only genes that showed a greater than 1.5-fold change were used in this analysis. The blue bars are the significance (-log (pvalue)), the orange line is the ratio given by number of significant genes in a pathway over number of genes in the pathway. **C:** Base read depth, scaled per million mapped reads, for *BMF* and *Tp63* genes in DMSO- or ACY-957-treated Karpas-422 cells. Screenshots taken from IGV are shown in the figure. **D:** ChIP analysis of H3K27ac, H3K27me3 and H3 at the promoters of *BMF* and *Tp63* genes was performed following treatment of Karpas-422 cells with either DMSO or 2μM ACY-957. Three independent ChIP experiments with H3K27me3, H3K27ac and H3 antibodies were done and the PCR was performed in triplicate. Histone H3 occupancy at a given target locus was used as a control to account for total histone levels. Rabbit IgG was also as a negative control to determine background signal. The threshold cycles from qRT-PCR (C_T values) obtained for the rabbit IgG control were between 31-33 cycles, and the signal obtained from using the H3K27ac or H3K27me3 antibodies ranged between 22-28. Hence, we obtained 8- to 512-fold enrichment with the H3K27me3 or the H3K27ac antibody in our ChIP assays when compared to the rabbit IgG negative control. To calculate fold change in the levels of H3K27 modification at a given target locus, we normalized the C_T value obtained from H3K27me3 or H3K27ac ChIP to the C_T value obtained from histone H3 ChIP. Fold-increase as determined by real-time PCR analysis in H3K27ac relative to total H3 occupancy was calculated for both ACY-957-treated and DMSO-treated samples and the relative fold-enrichment of H3K27ac and H4K27me3 in ACY-957-treated samples was calculated following normalization to DMSO-treated samples. The data represents an average fold-change calculated from three independent experiments +/- standard error. **p*=0.003 and ***p*=0.004 **E:** ChIP analysis of HDAC1 and HDAC2 at the promoters of *BMF* and *Tp63* genes was performed in Karpas-422 cells. Fold-increase as determined by real-time PCR analysis in HDAC1/HDAC2 relative to rabbit IgG occupancy was calculated from two independent experiments. The ChIP signal obtained from the antibodies recognizing HDAC1 or HDAC2 antibodies was normalized to the C_T values obtained from the input DNA. We then calculated fold enrichment in HDAC1 and HDAC2 ChIP signals relative to the negative rabbit IgG

control. This fold-change is depicted in panel E. Statistical analysis of the ChIP data was performed and p-values were calculated. *p=0.07, **p=0.0002, ***p=0.06 and ****p=0.003.

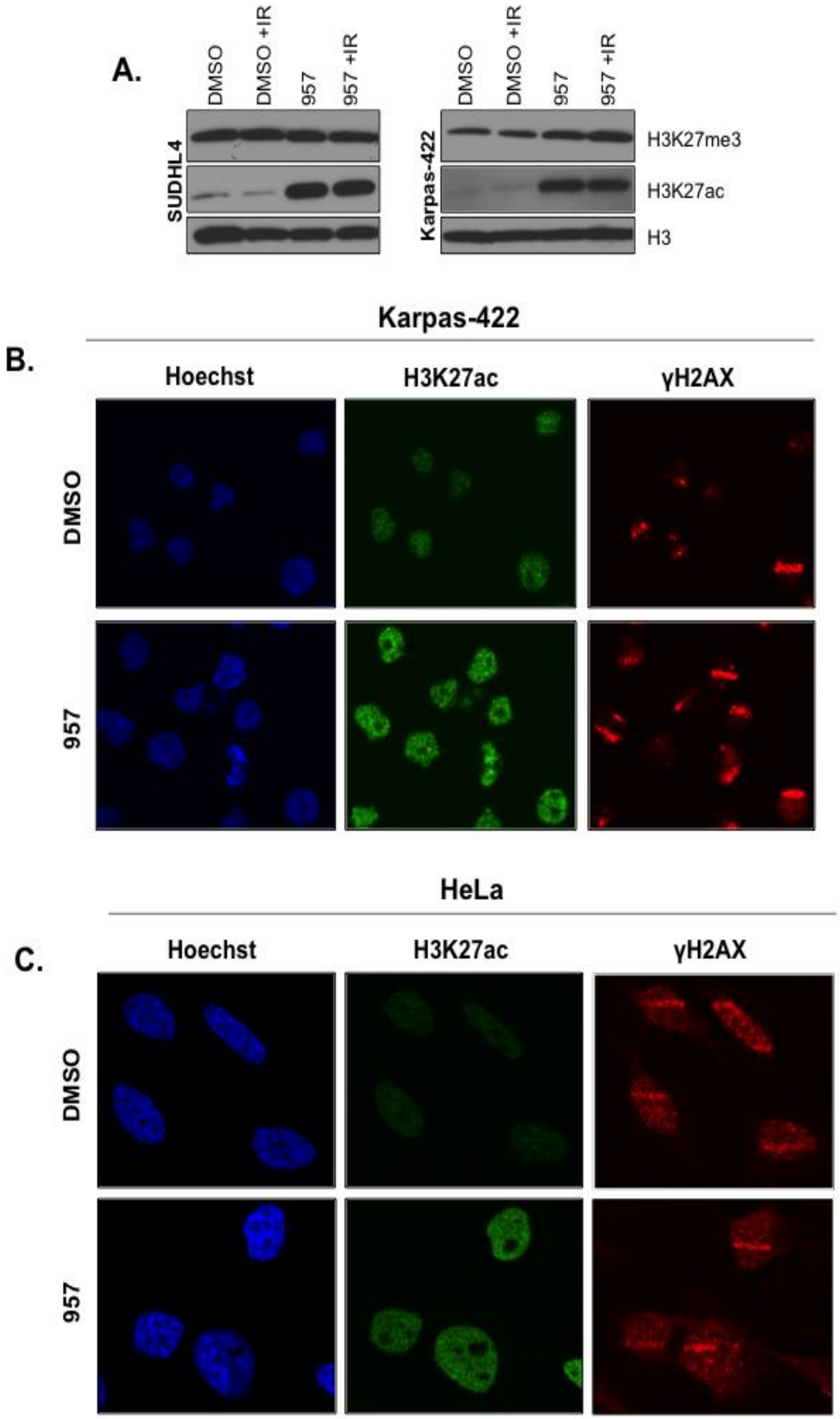
A.
DMSO
DMSO +IR
957
957 +IR
SUDHL4
Karpas-422
H3K27me3
H3K27ac
H3
Karpas-422
B.
Hoechst
H3K27ac
γH2AX
DMSO
957
HeLa
C.
Hoechst
H3K27ac
γH2AX
DMSO
957

Supplementary Figure 4: A: SUDHL4 and Karpas-422 cells were treated with DMSO or 2μM ACY-957 for 48h. Following DMSO or ACY-957 treatment, cells were exposed to a 5Gy dose of ionizing radiation and allowed to recover for 30 minutes prior to chromatin extraction. Western blots were performed with anti-H3K27ac or anti-H3K27me3 with histone H3 serving as a loading control. Karpas-422 (**B**) or HeLa (**C**) cells were micro-irradiated with a laser and allowed to recover for 15 minutes before fixation and immunofluorescence staining was performed with anti-γH2AX and anti-H3K27ac as described in the methods section.
